# Updated report on tools to measure outcomes of clinical trials in fragile X syndrome

**DOI:** 10.1186/s11689-017-9193-x

**Published:** 2017-06-12

**Authors:** Dejan B. Budimirovic, Elizabeth Berry-Kravis, Craig A. Erickson, Scott S. Hall, David Hessl, Allan L. Reiss, Margaret K. King, Leonard Abbeduto, Walter E. Kaufmann

**Affiliations:** 1 Departments of Psychiatry and Behavioral Sciences, Kennedy Krieger Institute and Child Psychiatry, Johns Hopkins University School of Medicine, 716 N. Broadway, Baltimore, MD 21205 USA; 2 Departments of Pediatrics, Neurological Sciences, Biochemistry, Rush University Medical Center, 1725 West Harrison, Suite 718, Chicago, IL 60612 USA; 3 Division of Child and Adolescent Psychiatry, Cincinnati Children’s Hospital Medical Center and the University of Cincinnati College of Medicine, 3333 Burnet Avenue MLC 4002, Cincinnati, OH 45229 USA; 4 Division of Interdisciplinary Brain Sciences, Department of Psychiatry and Behavioral Sciences, Stanford University, 401 Quarry Road, Stanford, CA 94305 USA; 5 MIND Institute and Department of Psychiatry and Behavioral Sciences, University of California Davis Medical Center, 2825 50th Street, Sacramento, CA 95817 USA; 6 Division of Interdisciplinary Brain Sciences, Departments of Psychiatry and Behavioral Sciences, Radiology and Pediatrics, Stanford University, 401 Quarry Road, Stanford, CA 94305 USA; 7 Autism & Developmental Medicine Institute, Geisinger Health System, Present address: Novartis Pharmaceuticals Corporation, US Medical, One Health Plaza, East Hanover, NJ 07936 USA; 8 MIND Institute and Department of Psychiatry and Behavioral Sciences, University of California Davis Medical Center, 2825 50th Street, Sacramento, CA 95817 USA; 9 Center for Translational Research, Greenwood Genetic Center, 113 Gregor Mendel Circle, Greenwood, SC 29646 USA; 10 Department of Neurology, Boston Children’s Hospital, Boston, MA 02115 USA

**Keywords:** Fragile X syndrome, Clinical trials, Outcome measures, Intellectual disability, Autism spectrum disorder

## Abstract

**Objective:**

Fragile X syndrome (FXS) has been the neurodevelopmental disorder with the most active translation of preclinical breakthroughs into clinical trials. This process has led to a critical assessment of outcome measures, which resulted in a comprehensive review published in 2013. Nevertheless, the disappointing outcome of several recent phase III drug trials in FXS, and parallel efforts at evaluating behavioral endpoints for trials in autism spectrum disorder (ASD), has emphasized the need for re-assessing outcome measures and revising recommendations for FXS.

**Methods:**

After performing an extensive database search (PubMed, Food and Drug Administration (FDA)/National Institutes of Health (NIH)’s www.ClinicalTrials.gov, etc.) to determine progress since 2013, members of the Working Groups who published the 2013 Report evaluated the available outcome measures for FXS and related neurodevelopmental disorders using the COSMIN grading system of levels of evidence. The latter has also been applied to a British survey of endpoints for ASD. In addition, we also generated an informal classification of outcome measures for use in FXS intervention studies as instruments appropriate to detect *shorter-* or *longer-term* changes.

**Results:**

To date, a total of 22 double-blind controlled clinical trials in FXS have been identified through www.ClinicalTrials.gov and an extensive literature search. The vast majority of these FDA/NIH-registered clinical trials has been completed between 2008 and 2015 and has targeted the core excitatory/inhibitory imbalance present in FXS and other neurodevelopmental disorders. Limited data exist on reliability and validity for most tools used to measure cognitive, behavioral, and other problems in FXS in these trials and other studies. Overall, evidence for most tools supports a *moderate* tool quality grading. Data on sensitivity to treatment, currently under evaluation, could improve ratings for some cognitive and behavioral tools. Some progress has also been made at identifying promising biomarkers, mainly on blood-based and neurophysiological measures.

**Conclusion:**

Despite the tangible progress in implementing clinical trials in FXS, the increasing data on measurement properties of endpoints, and the ongoing process of new tool development, the vast majority of outcome measures are at the *moderate* quality level with limited information on reliability, validity, and sensitivity to treatment. This situation is not unique to FXS, since reviews of endpoints for ASD have arrived at similar conclusions. These findings, in conjunction with the predominance of parent-based measures particularly in the behavioral domain, indicate that endpoint development in FXS needs to continue with an emphasis on more objective measures (observational, direct testing, biomarkers) that reflect meaningful improvements in quality of life. A major continuous challenge is the development of measurement tools concurrently with testing drug safety and efficacy in clinical trials.

## Background

Fragile X syndrome (FXS) is caused by an unstable expansion of a polymorphic trinucleotide (CGG) repeat sequence in the regulatory region of the fragile X mental retardation 1 gene (*FMR1*), which leads to its epigenetic silencing via atypical methylation [1]. This results in a deficit of the *FMR1*-encoded protein fragile X mental retardation protein (FMRP), an RNA-binding protein that regulates dendritic translation and plays a critical role in synaptic development and function [1, 2]. With a prevalence of about 1/4000, FXS is a relatively common single monogenic cause of intellectual disability (ID) and autism spectrum disorder (ASD). Patients with FXS experience a wide range of cognitive, behavioral, and systemic manifestations that often require a complex array of behavioral and pharmacological interventions [2, 3]. A number of studies of symptom-based pharmacological treatments in individuals with FXS have been performed in small cohorts or without randomization or blinding (reviewed in [4]). Thus, as for other neurodevelopmental disorders with behavioral symptoms, guidance is somewhat lacking regarding the effectiveness of targeting clinical manifestations of FXS [e.g., attention-deficit hyperactivity disorder (ADHD)]. Nonetheless, advances in experimental models of FXS and other neurodevelopmental disorders with known genetic bases have opened the door for the development of disease- and neurobiological mechanism-specific pharmacological treatments [5].

From the beginning of the era of neurobiological “targeted” trials in FXS, there have been concerns about endpoints. Thus, the National Institutes of Health (NIH) convened two meetings of leading scientists and clinicians, which included other key stakeholders (e.g., Food and Drug Administration (FDA), advocacy groups, pharmaceutical industry) to discuss the subject of outcome measures. The second and key meeting, held in 2009, led to the creation of Outcome Measures Working Groups (here termed Working Groups) and a publication summarizing the findings of these Groups [6]. Problems identified during these meetings included the following: (1) few measures were validated for FXS; (2) investigators used different measures due to lack of consensus; (3) measures were unable to cover multiple levels of function; (4) limited number of measures were validated or standardized for ID populations, with many having substantial floor effects; (5) measures involving direct observation were lacking (i.e., most instruments were rating scales); and (6) biomarkers that quantify brain function, viewed as the most useful biomarkers, had demonstrated clear differences between FXS and control individuals but none had well-established clinical correlations or validity [6]. To address these shortcomings, at the 2009 meeting, the Working Groups were tasked with identifying outcome measures in three areas relevant to the broad phenotype of FXS: (1) Cognition, (2) Behavior/Emotion, and (3) Medical/Physical including biomarkers. Their work was guided by three principles: (1) identification of a core set of widely applicable measures would facilitate comparability across different agents, research centers, and methodological approaches; (2) outcome measures should be validated specifically for FXS since those that have been developed for symptom-based clinical trials in behaviorally defined disorders (e.g., ADHD) might not be sufficiently sensitive or specific; and (3) the Working Groups should generate a set of criteria for identifying appropriate outcome measures for design, interpretation, and guiding funding of clinical trials in FXS [6]. In addition to specific recommendations for each of the three areas, the Working Groups concluded that (1) research on biomarkers for detecting treatment response in FXS was in its infancy, but this was an area of utmost importance; (2) measures under development have to be linked to the neurobiology of FXS; and (3) continued research is greatly needed in multiple areas, including the core behavioral field.

Since 2009, there has been an explosion of clinical trials in FXS, especially targeted drug interventions (reviewed in [7]). Despite adequate power and other study design strengths, successful phase II trials that advanced to phase III failed to demonstrate therapeutic efficacy based on their primary endpoints [8, 9]. This has raised questions about the promise of translating breakthrough drug studies in mouse and other experimental models to humans with FXS. Although the ongoing analysis of methodological issues underlying these failures has identified multiple possible explanations, inadequacy of available biomarkers and outcome measures is unquestionably a factor [8]. This situation has led to a re-assessment of the quality of existing outcome measures in FXS and the need to update the recommendations from the Working Groups in the 2013 Report [6]. The perceived “crisis” in the FXS treatment field also represents an opportunity to implement novel study designs and methodologies. An example of this is an upcoming trial re-examining the efficacy of the metabotropic glutamate receptor 5 (mGluR5) antagonist mavoglurant in FXS, which will introduce modifications such as the study of younger individuals, presumably more receptive to interventions targeting synaptic development, and the use of a cognitive intervention during the drug trial, aimed at enhancing drug effect on learning and consequently on synaptic plasticity. Introduction of novel outcome measures and examination of potential biomarkers already studied in mouse models (e.g., auditory evoked potentials) are also components of this innovative trial (U01 NS096767, Effects of AFQ056 on Language Learning in Young Children with Fragile X Syndrome, P.I. Berry-Kravis).

This report represents a re-assessment of outcome measures in FXS taking into account progress in the field since the 2013 publication. It preserves the principles (i.e., critical review and recommendations for measures currently available or under development) and structure (i.e., Cognition, Behavior/Emotion, Biomarkers/Medical types of outcome measures) of the 2013 Report. Nevertheless, it does also introduce new elements. These include the classification of measures in terms of their projected ability to detect *shorter-term* and/or *longer-term* changes and a grading of endpoints based on their measurement/psychometric properties using the COnsensus-based Standards for the selection of health Measurement INstruments (COSMIN) system (www.cosmin.nl). The latter was recently applied by the UK NHS’ National Institute for Health Research to the evaluation of tools measuring outcomes in young children with ASD [10–12]. We hope this re-evaluation of outcome measures in FXS will provide guidance for the design and implementation of clinical trials (pharmacologic or other interventions) in this and other neurodevelopmental disorders.

## Material and methods

The basis for our evaluation of available outcome measures was an extensive database search that included MEDLINE/PubMed, Embase, PsycINFO, and Web of Science, originally performed in December 2015 and rerun in January 2016. The search used keywords for individual outcome measures, including their reliability or validity in FXS, in general, and in clinical trials, in particular. For example, “ADI-R” AND “fragile X” OR “ADI-R” AND “fragile X” AND “reliability,” OR “ADI-R” AND “fragile X” AND “validity”. If the data were limited or not available for FXS, an additional search included other fields of relevance (e.g., ASD, Rett syndrome). For instance, “ADI-R” AND “autism spectrum disorder” OR “ADI-R”“AND “autism spectrum disorder” AND “reliability,” OR “ADI-R” AND “autism spectrum disorder” AND “validity”. In addition to these systematic searches, several authors applied their experience with the use or development of certain measures for trials involving FXS or other neurodevelopmental disorders.

After dividing Cognition, Behavior/Emotion, and Biomarker/Medical (Medical/Physical in the 2013 Report) outcome measures into distinct subdomains, we generated an expert consensus on their classification as *shorter*- and/or *longer-term* outcome/effect endpoints. An initial criterion for the *shorter-term* grouping was experience with the measures in FXS placebo-controlled trials lasting less than 12 months (see Table 1). On the other hand, a measure was considered as *longer-term* outcome on the basis of its use in open-label extensions of controlled clinical trials, the majority of them lasting 12 months or longer [7]. Due to limited empirical evidence, these criteria were complemented by expert agreement on particular types of function and the time necessary for detecting change. Because of their temporal dimension, typically at least 1 year, *longer-term* outcomes tend to reflect more global levels of function in particular in the cognitive domain (e.g., crystallized knowledge evaluated by intelligence and academic skills tests, adaptive behavior skills). Using the same logic, some types of manifestations, particularly problem behaviors, could change over short or long time periods. Thus, most behavioral measures could be either *shorter*- or *longer-term* outcomes. We considered *shorter-term* outcome measures those that capture functions that are more fluid and have the potential to change rapidly. For example, in the cognitive domain, working memory and fluency and some attentional aspects.

**Table 1:** Double-blind, placebo-controlled treatment studies of individuals with fragile X

FDA register number	Compound	*Mechanism*	Cohort (years)	Sample size	Outcomes measured (OM)		Phase/sponsor	Results/publications
					Primary^1^	Secondary^2^		
		*14/22, 64%*						
NCT00788073	Arbaclofen (STX209)	*GABA-B agonis*t	6–50	63	ABC-C_I	ABC-C_FX_, CGI-S, CGI-I, Vineland-II, SRS, RBANS, SB5, PPVT	II/Seaside	OM:^1^ not met, ^2^↓ABC-C_FX_-SA [112]
NCT01282268	Arbaclofen	*GABA-B agonist*	12–50	125	ABC-C_FX__SA	CGI-S, CGI-I, Vineland-II, ADHD-RS, SB5, PSI, CSHQ	III/Seaside	OM:^1^ not met [46]
NCT01325220	Arbaclofen	*GABA-B agonist*	5–11	172	ABC-C_FX__SA	CGI-S, CGI-I, Vineland-II, ADHD-RS, SB5, PSI, CSHQ	III/Seaside	OM:^1^ not met, ^2^↓ABC-C_FX_-I, ↓PSI [46, 113]
NCT01725152	Ganaxolone	*GABA-A agonist*	6–17	60	CGI-I	ABC-C_FX_, ADAMS, PPI, KiTAP, ERP, ET, SNAP-IV, PARS	II/Marinus	Pending
NCT02126995	Metadoxine	*GABA transporter*	14–50	62	ADHD-RS	ABC-C_FX_, CGI-S, CGI-I, Vineland-II, SRS, KiTAP, ERP, ET, PARS	II/Alcobra	OM:^1^ not met, ^2^↑Vineland II DL [44, 64]
NCT00718341	Mavoglurant (AFQ056)	*mGluR5 antagonist*	18–35	30	ABC-C_T	ABC-C_FX_, CGI-S, CGI-I, SRS, KiTAP, ET, VAS, RBANS..	II/Novartis	OM:^1^ not met,^2^ ↓ABC-C_T (*n*=7 FM) [261]
NCT01253629	Mavoglurant	*mGluR5 antagonist*	18–45	175	ABC-C_T	ABC-C, CGI-S, CGI-I, SRS, KiTAP	IIb/Novartis	OM:^1^ & ^2^ not met, tolerated well [9]
NCT01357239	Mavoglurant	*mGluR5 antagonist*	12–17	139	ABC-C_T	ABC-C, CGI-S, CGI-I, SRS, KiTAP	IIb/Novartis	OM:^1^ & ^2^ not met, tolerated well [9]
NCT01015430	Basimglurant (RO4917523)	*mGluR5 antagonist*	18–50	40	Safety, PK	ABC-C, ADAMS, CGI-S, CGI-I, KiTAP, ET, VAS, RBANS	IIa/Roche	Not in public domain
NCT01517698	Basimglurant	*mGluR5 antagonist*	14–40	185	ADAMS_T	ABC-C, ADAMS, CGI-S, CGI-I, WISC-IV, CBI-M	IIb/Roche	Not in public domain
NCT01750957	Basimglurant	*mGluR5 antagonist*	5–13	180	Safety	PK, ABC-C, ADAMS, CGI-S, CGI-I, WISC-IV, CBI-M	IIb/Roche	Not in public domain
NCT01911455	Acamprosate	*GABA/Glutamate*	5–23	48	ABC-C_SW	CGI-S, CGI-I, VAS, ABC, PARS, ADAMS, CSQ, ELS, SRS	II/III Cinn Child	Pending
NCT01894958	Trofinetide	*IGF-1 analog*	14–40	70	Safety	CGI-S, CGI-I, ABC-C_FX_, SRS, FXS scale, VAS-P/C, KiTAP	II/Neuren	OM: ﻿^1^Tolerated well, ^2^-several improved [63]
NCT00054730	CX516	*Ampakine*	18+	49	Memory	CGI-I, multiple *Z* scores, SNAP-IV	II/Cortex	OM:﻿ ﻿^1^ Tolerated well,^2^↑CGI-I in a subset [40]
NCT01053156	Minocycline	MMP-9	3–16	66	CGI-I,VAS	VAS-mb, ABC-C, Vineland-II, EVT-2	II/UC Davis	OM:^1^ ↑CGI-I, ^2^↑VAS, ↓MMP-9; [87]
NCT01474746	Sertraline	SSRI	2–6	30	CGI-I,VAS	Vineland-II, PSI-4, PLS-5, MSEL	II/UC Davis	OM:^1^↑CGI-I (46%),^2^ ↑VAS [262]
NCT01120626	Donepezil	Cholinergic drug	12–29	45	ABC-C_T/CNT	MRS, WM tests, CARS	II/Stanford	Pending
NCT01254945	Oxytocin	Brain peptide	13–28	8	Eye gaze	HR, RSA, HRV, cortisol	Ib/Stanford	OM:^1^↑Eye gaze, ^2^↓cortisol [77, 263]
NCT01329770	Vitamins C & E	Antioxidants	6–18	30	DBC, WISC	Tolerability	II/Imabis	Pending [264]
N/A	Melatonin	Hormone	2–15	15	Sleep times	Actiwatch	II/UC Davis	OM:^1^↑sleep; [265]
N/A	L-AcCarnitine	Brain energy	6–13	63	ConnersGI-P	Vineland-Survey, WISC-R	II/TELETHON	OM:^1^↓HI/I, ^2^↑Vineland [266]
N/A	MPH/DAMP	Stimulants	3–11	15	ConnersAbb	ACTeRS, IQ measures	II/Denver Child	OM:^1^MPH:↓I, ^2^↓ACTeRS; [267]

Modified from Table 1, Budimirovic and Phan [268] with permission

*Abbreviations: FDA* Federal Drug Administration, *GABA* gamma-aminobutyric acid, *ABC-C_I* Aberrant Behavior Checklist-Community Edition (ABC-C), Irritability subscale, *CGI-S* Clinician Global Impression-Severity, *CGI-I* CGI improvement, *VABS-II* Vineland Adaptive Behavior Scale, Second Edition, *SRS* Social Responsiveness Scale, *RBANS* Repeatable Battery for the Assessment of Neurological Status, *SB5* Stanford-Binet Version 5, *ABC-C*_*FX*_ *_SA* ABC-C_FX,_ Social Avoidance subscale, *PSI* Parenting Stress Index, *CSHQ* Children’s Sleep Habits Questionnaire, *PPVT* Peabody Picture Vocabulary Test, *ADHD-RS* Attention-Deficit Hyperactivity Disorder Rating Scale, *ABC-C*_*FX*_ ABC-C, FXS-specific, *ADAMS* Anxiety Depression and Mood Scale, *PPI* prepulse inhibition, *KiTAP* Tests of Attentional Performance for Children, *ERP* evoked response potentials, *ET* eye tracking, *SNAP-IV* Swanson, Nolan and Pelhram Rating ﻿Scale, *PARS* Pediatric Anxiety Rating Scale, *ABC-C_T* ABC-C total score, *ADAMS_T* ADAMS total score, *WISC-IV* Wechsler Intelligence Scale for Children, 4th ed, *CBI-M* Caregiver Burden Inventory-Modified, *MMP9* matrix metallopeptidase 9, *VAS* visual analog scale (*VAS-C*, clinician rated; *VAS-mb*, multiple behaviors; *VAS-P*, parent rated), *mGluR5antagonist* metabotropic glutamate receptor 1/5 antagonist, *GABA/Glutamate* GABA/glutamate normalizer, *ABC-C_SW* ABC-C, Lethargy/Social Withdrawal subscale, *CSQ* Caregiver Strain Questionnaire, *ELS* Expressive Language Sampling, *Trofinetide* Neu-2566-FXS-001 study, *IGF-1* insulin-like growth factor, *PLS-5* Preschool Language Scale-Version 5, *MSEL* Mullen Scales of Early Learning, *CNT* Contingency Naming Task, *MRS* magnetic resonance spectroscopy, *WM* working memory tests, *CARS* Childhood Autism Rating Scale, *HR* heart rate, *RSA* respiratory sinus arrhythmia, *HRV* heart rate variability, *SSRI* selective serotonin reuptake inhibitor, *DBC* Developmental Behavior Checklist, *EVT-2* Expressive Vocabulary Test, 2nd ed, *PK* pharmacokinetics, *ConnersGI-P* Conners General Index-Parent Version, *WISC-R* WISC-Revised, *MPH* methylphenidate, *DAMP* dextroamphetamine, *Conners Abb* Conners Abbreviated Version, *ACTeRS* ADD-H Comprehensive Teacher’s Rating Scales, *IQ* intellectual quotient

^1^reflects a primary outcome measure (OM)

^2^reflects a secondary outcome measure (OM)

*Italized* under M﻿echanism are clinical trials targeting excitatory-inhibitory imbalance in FXS﻿

After determining the duration of outcomes for each measure, we graded tool quality [11, 13–15]. Three criteria were used: (1) whether reliability and validity data was available in typically developing individuals and/or in neurodevelopmental disorders (ID, ASD, FXS, Rett syndrome), (2) whether the measure had been piloted in FXS clinical trials (Table 1; [4]), and (3) whether the measure addressed an important aspect of the FXS phenotype [2, 16]. The grading was done using the COSMIN system that includes the following levels (from best to worst): *strong*, *moderate*, *limited*, *unknown due to poor methodological quality*, and *no evidence available*. Adapting these grades to our criteria, a strong (“+++”) label required evidence of (1) compelling reliability and validity (e.g., excellent internal consistency) in typical individuals and/or several neurodevelopmental disorders, including FXS, and (2) having been piloted in three or more FXS clinical trials that showed sensitivity to treatment. A moderate (“++”) label required evidence of (1) acceptable reliability and validity data (e.g., good internal consistency) in typical individuals and/or one or more neurodevelopmental disorders, including FXS, and (2) being piloted in one or two FXS clinical trials. The limited (“+”) label was considered if there was (1) weaker evidence of reliability and validity (e.g., fair internal consistency) in typical individuals and/or one neurodevelopmental disorders and (2) no experience in FXS clinical trials. The unknown due to poor methodological quality and no evidence available labels differed in that the former had some data on reliability or validity and/or the available data was of poor methodological quality.

In order to illustrate the psychometric properties we used for determining the tool quality of each instrument, here, we provide some quantitative parameters employed in psychometric assessments. Internal consistency, typically measured by the Cronbach’s alpha coefficient, is evaluated by the following interpretative guidelines: excellent (≥0.90), good (0.80–0.89), fair (0.70–0.79), and unacceptable (<0.70). Multiple measures of reliability (e.g., test-retest, inter-rater) are usually evaluated by intraclass correlation coefficients as follows: excellent (≥0.75), good (0.60–0.74), fair (0.40–0.59), and poor (<0.40). For more information about psychometric evaluations, we recommend the reader our recent publication on anxiety measures in Rett syndrome [17] as well as the UK’s NHS National Institute for Health Research comprehensive review of behavioral outcome measures for young children with idiopathic ASD [11]. Details about measurement properties of each instrument can be found in the key original publications, all included in the “References” section.

## Results

The period between 2008 and 2015 saw tangible progress in controlled clinical trials in FXS, particularly those applying neurobiological-targeted treatments. Table 1 depicts studies in individuals with FXS that employed best practices methodology (randomized, double-blind, placebo-controlled trials). To date, a total of 22 such trials have been identified through search of literature and other sources; 19/22 (86%) have been registered on the www.ClinicalTrials.gov website, and data on more than half of the studies have been published to date.

As expected from FXS’s neurobiology, the vast majority of the studies have targeted a core excitatory/inhibitory imbalance in the disorder (italized under M﻿echanism, Table 1) primarily through either mGluR5 antagonists (mavoglurant-AFQ056, basimglurant-RO4917523) or GABA agonists (arbaclofen-GABA-B agonist, ganaxolone-GABA-A agonist, acamprosate-GABA-A/GABA-B agonist). These studies represent the majority of the total (14/22, 64%) and registered (14/19, 74%) trials. Reflecting that over two thirds of these trials were phase II, most of them have studied adults and adolescents (i.e., FDA recommendations indicate that novel drugs should be tested first in adults, particularly in vulnerable populations). Some trials targeted other glutamatergic components (AMPA receptors: ampakine CX-516) or more general synaptic mechanisms (IGF-1 tripeptide analog trofinetide, minocycline). The rest of the reported studies focused on other systems presumably affected by FMRP deficit: serotonergic and cholinergic drugs, the “social brain” neuropeptide oxytocin, compounds that affect metabolism, and other modulators.

Experience from these trials was used to re-assess the quality of outcome measures employed. Based on the latter data and the literature, the following sections re-evaluate the utility and quality of available endpoints, grouped into the three categories used in the 2013 Report: Cognition, Behavior/Emotion, and Biomarker/Medical. Evidence and recommendations for each area are outlined as follows: *Background*, *Progress and plans in FXS*, *Potential measures*, and *Conclusions.* Due to the large number and diversity of measures, the Behavior/Emotion and Biomarker/Medical domains are divided into subdomains.

## Cognitive measures

### Background

Individuals with FXS present with variable cognitive, adaptive, and language impairments. Boys with FXS who do not meet the criteria for ASD typically function in the mild to moderate ID range (~FSIQ 40–70), whereas those with ASD are often lower functioning [18, 19]. Expressive language is typically more affected than receptive language in individuals without ASD. Since cognitive impairments are core FXS phenotypes, it is postulated that most therapeutic interventions targeting key mechanisms related to FMRP deficit (e.g., mGluR5 antagonists, trofinetide) would have an impact on global or specific cognitive domains, including communication abilities [20, 21]. Most measures of cognition have been employed in observational studies, even after the beginning of the clinical trial era in FXS. The 2013 Report recommended (1) validation of an expressive language sampling technique to measure improvements in language described anecdotally by parents during clinical trials and (2) identification of measures of cognition and executive function that can be performed by individuals with FXS and for which floor effects can be avoided. The Working Group also recommended that, although promising measures were identified for several key domains, further data were needed on several properties before their use for clinical trials: (1) test-retest reliability, sensitivity, and validity; (2) validation over an expanded age range; and (3) feasibility and validity across the full range of affectedness. Here, we review measures of global cognitive function, including adaptive behavior skills, followed by instruments used for evaluating selective cognitive functions (Table 2).

**Table 2:** Global and other cognitive domains and outcome measures in fragile X syndrome

Domain	Outcome measure	*Shorter term*	*Longer term*	Quality of tools
Intellectual quotient	Wechsler_WAIS-IV, WPPSI-IV, WISC-V		Yes	++ [23–25, 32, 33]
	Stanford-Binet 5		Yes	++ [22, 33, 34, 46, 112]
Multiple cognitive skills	NIH Toolbox Cognitive Battery	Yes^a^	Yes	+ [35–38]
Adaptive behavior				
	Vineland-II		Yes	++/+++ [19, 29, 44, 46–49, 98, 112]
	ILS		Yes	+ [97–99]
	W-ADL		Yes	+ [100]
Memory & Learning				
	WJ working memory	Yes	Yes	+ [50, 51]
	RBANS	Yes	Yes	+/++ [53–56]
	SB-5 Block Span	Yes	Yes	+ [22, 33]
Executive function				
	KiTAP (4 subtests)	Yes	Yes	++/+++ [44, 46, 60–63, 112]
	WJ auditory attention	Yes	Yes	+ [50, 51]
	WJ digits reversed	Yes	Yes	+ [50, 51]
Language				
	PPVT-4		Yes	+ [66, 67, 72–74]
	EVT-2		Yes	+ [68]
	CELF-5/P2		Yes	+ [66, 69, 70]
	FMP	Yes		+ [72–75]
	ELS	Yes	Yes	++ [6, 21, 44, 76]
Social cognition				
	SRS	Yes	Yes	+/++ [15, 17, 46, 78, 80, 81, 112, 113]
	Eye tracking	Yes	Yes	++ [85, 87, 88]
	SPPA	?	?	? [89]
Academic achievement				
	WJ Achievement III		Yes	? [90–93]
	WIAT-II		Yes	

*Abbreviations*: *Wechsler_WAIS-IV* Wechsler Adult Intelligence Scale, 4th ed, *WPPSI-IV* Wechsler Preschool and Primary Scale of Intelligence, 4th ed, *WISC-V* Wechsler Intelligence Scale for Children, 5th ed; Stanford Binet5, 5th edition, *NIH* National Institute of Health, *Vineland-II* Vineland Adaptive Behavior Scale-2nd Ed, *ILS* Independent Living Scales, *W-ADL* Waismen Activities of Daily Living Scale, *WJ* Woodcock Johnston, *RBANS* Repeatable Battery for the Assessment of Neurological Status, *KiTAP* Test of Attentional Performance for Children, *PPVT-4* Peabody Picture Vocabulary Test-4th ed, *EVT-2* Expressive Vocabulary Test-2nd ed, *CELF-5/P2* Clinical Evaluation of Language Fundamentals-5th edition/Preschool-2nd ed, *FMP* fast-mapping performance, *ELS* expressive language sampling, *SRS* Social Responsive Scale, *SPPA* Self-Perception Profile for Adolescents, *WIAT-II* Wechsler Individual Achievement Test-II. *Shorter term* deemed suitable for clinical trials lasting <12 months; *Longer term* deemed suitable for clinical trials lasting >=12 months. Grading system: *+++* strong evidence, *++* moderate evidence, *+* limited evidence, *?* unknown due to poor methodological quality, *blank cell* no evidence available

^a^Yes applies for some subtests

### Progress and plans in FXS

1. Global cognitive measures. These include tests of general intelligence and adaptive behavior skills. The former yield IQ scores, including the Stanford-Binet Intelligence Scales (Fifth Edition, SB5) [22] and the Wechsler Intelligence Scales [23–25], and developmental scales that measure cognition in young children such as the Mullen Scales of Early Learning [26] and the Bayley Scales of Infant Development [27]. These instruments and adaptive behavior scales have been long used in FXS in the context of a variety of observational studies [28, 29], in particular the Vineland Adaptive Behavior Scales [30], now in its 3rd Edition (Vineland-3; [31]).
  - General intelligence tests. While these cognitive measures have good psychometric properties, IQ tests have known floor effects when applied to individuals with significant cognitive impairment (i.e., below mild ID level), limiting their sensitivity to change, group and individual differences, and relative strengths and weaknesses within the individual. Hessl and colleagues have developed scoring modifications that address these shortcomings and allow better estimates of profiles of cognitive strength and weakness [32, 33]. The latest publication, which developed “deviation scores” for the SB-5 in individuals with FXS and idiopathic ASD with comorbid ID [33], suggests that the SB-5, with a very broad developmental range, may be an adequate *longer-term* outcome measure for tracking developmental progress in FXS trials. Altogether, the evidence of progress on the use of the SB-5 in FXS places it at the *moderate* tool quality level. As discussed in the adaptive behavior section, it is important to know the developmental trajectories of global cognitive measures. In general, IQ scores tend to decrease with age during childhood, reflecting a widening gap or slower progress in individuals with FXS compared to normal cognitive development. In fact, Quintin and colleagues, using the deviation method described above, showed in a group of 184 children and adolescents with FXS, aged 6–16 years, that the cognitive profile associated with FXS develops dynamically from childhood to adolescence and varies depending on cognitive domain [34]. Therefore, careful interpretation of changes of standard scores with age is needed, and the use of special scores that are not age-adjusted may be useful in detecting change in clinical trials (e.g., SB-5 change-sensitive scores).
    The NIH Toolbox Cognitive Battery (NIH-TCB) is a web-based and touch-screen response neuropsychological tool normed on children and adults [35], with a primary focus on its use as a series of cognitive endpoints such as executive function, attention, working memory, processing speed, episodic memory, single-word reading, and receptive vocabulary for a variety of populations and research questions [36]. It yields fluid reasoning, crystallized, and overall cognition composite standard scores analogous to the IQ scores described above. The battery is available in both English and Spanish, with recently demographically corrected normative standards for the English version [37]. The primary goal of the NIH-TCB is to employ a more efficient, common metric for cross-study comparisons, but it does not substitute in-depth, comprehensive neuropsychological batteries [36]. Piloting and validation of the NIH-TCB in populations with ID (i.e., FXS, Down syndrome, and other forms of ID) to determine feasibility, reliability, validity, and sensitivity to change (after 2 years) are in progress as a four-site NICHD-funded project (1R01HD076189) awarded in response to the PAR-13-213, Outcome Measures for Use in Treatment Trials for Individuals with Intellectual and Developmental Disabilities (PIs: Hessl, Berry-Kravis, Riley, Gershon). A recently published series of pilot studies from this group using NIH-TCB measures provides preliminary evidence of feasibility, test-retest reliability, convergent validity, and sensitivity to group differences [38]. In theory, the NIH-TCB could be used as either a *shorter*- or *longer*-*term* outcome measures; we grade it as a *limited* quality tool until the complete study results with larger sample sizes become available and utility is also confirmed. More information about measurements of specific cognitive functions is provided below in sections 2–5.
  - Adaptive behavior scales. This is a behavioral domain closely linked to cognition, representing to large extent the application of cognitive function to daily life. Thus, the Diagnostic and Statistical Manual for Mental Disorders-5^th^ Edition (DSM-5)’s definition of ID places adaptive skills at the center of intellectual functioning [39]. Most recent data on adaptive skills in FXS has been obtained with the Vineland-II, which has been validated in children and adults with ID and used in populations such as ASD, FXS, Down syndrome, Angelman syndrome, and Rett syndrome [17, 40–43]. Despite this, the Working Group found that few adaptive behavior measures had been validated or standardized for populations with ID, and most had significant floor effects. The Vineland-II has been employed as a secondary outcome measure in three recent FXS clinical trials. In a randomized, double-blind, placebo-controlled, multi-site phase IIb trial of metadoxine (MDX, an ion pair salt of pyridoxine or vitamin B6 and L-pyroglutamate, Alcobra) in FXS (AL014 trial) (Table 1), involving 62 adolescents and adults, aged 14–50 years, Berry-Kravis reported that individuals with FXS gained an average of 2.6 standard score points on the Vineland-II Daily Living Skills domain following 6 weeks of treatment (vs. 1.4 standard score points in the placebo group; *p* = 0.035) [44]. Additional analyses indicated that the effect was greater in younger individuals (<18 years) and those with higher, although not normal IQ. These results suggest that the Vineland-II Daily Living Skills domain may be sensitive to drug effects over a relatively short period of time. In two phase III trials of arbaclofen in males and females with FXS (STX209), a flexible dose trial involving 119 patients, aged 12–50 years, and a fixed dose trial involving 159 patients, aged 5–11 years, no improvements were found on the Vineland-II Socialization. On the other hand, it was also reported that some patients derived benefit from arbaclofen on the Vineland-II Socialization subscale following a long-term open-label extension [45, 46].
    Several recent studies have documented the extent to which standard scores on the Vineland-II may deviate relative to the normative data at particular age bands in FXS. For example, in a cross-sectional study of boys with FXS, standard scores on the Vineland-II declined between 1 and 4 points per year depending on the subdomain [47]. Similarly, in a large-scale longitudinal study involving 275 males and females with FXS, aged 2–18 years, Klaiman and colleagues reported that standard scores for males declined by approximately 3.3 points per year on average on the Vineland-II Composite, by 4.5 points per year on the Daily Living Skills domain, by 3.5 points per year on the Communication domain, and by 2.8 points per year on the Socialization domain [29]. Declines in standard scores with age were smaller in females and lessened as the subjects aged into adolescence. Interestingly, a recent longitudinal study involving 47 boys with FXS, between 9 and 15 years, reported that standard scores on the Vineland-II Socialization and Communication domains improved with age whereas scores on the Daily Living Skills remained relatively flat [48]. This indicates that the development of some adaptive behavior domains may be relatively less affected than others; however, more data are needed to clarify this age and gender differences.
    Note: Decline in standard scores of cognitive or adaptive behavior measures with age reflects the fact that affected individuals are not keeping pace with the normative typically developing samples, rather than reflecting any loss of adaptive skills.
    Altogether, these data indicate that the Vineland-II is a suitable instrument for *longer*-*term* studies (e.g., 1 year in length); however, these investigations will need to factor the natural decline or increase in scores at particular age bands. Strengths of the Vineland-II as a *longer*-*term* outcome measure include its low floor of 20 standardized points, ease of administration (parent interview), and relatively short administration time (~1 h). Caveats of the measure include the potential for reporting bias, low sensitivity to change, and the fact that the psychometric properties of this instrument are unknown in the FXS population (although one study has reported that the internal consistency of the Vineland-II in individuals with ID is high [49]). At present, the Vineland-II should be considered a measure of *moderate* tool quality. Although no data are yet available, early application of the Vineland-3 [31] to FXS indicates a similar performance to the Vineland-II.
2. Memory and learning. The Working Group identified several memory tasks that mapped onto the FXS memory phenotype, such as the Auditory Working Memory and the Digits Reversed subtests of the Woodcock-Johnson III Tests of Cognitive Abilities [50, 51], the Corsi Blocks [52], and the SB-5 Block Tapping component of Visual Working Memory (visual-spatial sequential memory). While no published data are available on reliability or validity in subjects with FXS to determine their quality, these memory tasks have the potential of being used as either *shorter*- or *longer*-*term* outcome measures. The Working Group also proposed assessing long-term memory with the List Learning and Story Memory subtests of the Repeatable Battery for the Assessment of Cognitive Status (RBANS; [53, 54]). The RBANS is a neuropsychological assessment that covers five domains (Immediate Memory, Language, Attention, Visuospatial/Constructional, Delayed Memory), developed in part as a neuropsychological screening battery for adult patients with neurological disorders. The List Learning subtest showed significant improvement in an open-label trial of lithium in 15 individuals with FXS, aged 6–23 years [54, 55]. A placebo-controlled trial of the ampakine CX516 in 49 patients with FXS, aged 18–49 years, did not find a significant improvement in their cognition based on composites of several subtests including primarily the RBANS; however, some of the RBANS subtests showed high feasibility and test-retest reliability (List Learning, Story Memory, List Recognition, Digit Span) [40]. Administration of the RBANS via video teleconference remotely was found to be feasible and reliable when compared to face-to-face administration [56]. Thus, the RBANS could be used as either a *shorter*- or *longer*-*term* outcome measure, showing preliminary evidence of reliability and validity (and possibly sensitivity to change). This places the RBANS in the *limited*-*moderate* tool quality.
3. Executive functioning (EF). There is compelling evidence that deficits in EF, present in most neurodevelopmental disorders, are highly characteristic of individuals with FXS [57, 58] with performance often below mental age expectations [59]. The Working Group identified several measures of EF that appear to be well suited for use in clinical trials (i.e., KiTAP 4 subtests, Woodcock-Johnson Rapid Naming). Nonetheless, some of these measures may be beyond the ability of lower-functioning individuals.
  - KiTAP. This is a set of computer-based assessments of EF, which was standardized for typically developing children, ages 6–10 years [60]. The KiTAP is composed of eight tests that vary in length and difficulty level, capturing attention, inhibitory control, and cognitive flexibility [61]. An advantage of the KiTAP for FXS is the visual nature of the tasks, which taps into a relative strength. A pilot validation study of the KiTAP in FXS, including 36 males and females, aged 7–50 years, identified four subtests that generate measures of EF with good feasibility, reproducibility, and minimal ceiling and floor effects [62]. These four specific subtests were Alertness reaction time, Distractibility commission errors, Go/No-Go commission errors, and Flexibility errors, all of which demonstrated good clinical validity [i.e., correlation with Attention or Hyperactivity subscales of the Aberrant Behavior Checklist-Community (ABC-C) or the Behavioral Assessment for Children Scale (BASC)] and test-retest reliability [62]. All these features led to the inclusion of the KiTAP in five clinical trials. The KiTAP could be used as a *shorter*-*term* outcome measure, with psychometric evidence supporting a *moderate* to *strong* tool quality label, as we wait more data on sensitivity to treatment (i.e., [63], Table 1). In the recent phase II trial of metadoxine mentioned in the previous section on adaptive behavior, scores on the KiTAP Go/No-Go subscale improved significantly with a relatively large effect size [44, 64]. EF measures in the NIH-TCB are currently under investigation.
    The Working Group also suggested the use of informant report measures (e.g., the Behavior Rating Inventory of Executive Function or BRIEF [65]), which may prove useful when direct assessment is not possible. However, a recent pilot study aiming to validate the NIH Toolbox Cognitive Battery for ID, including those with FXS, showed weak correlations between caregiver-reported EF on the BRIEF and the objectively measured similar constructs of EF (attention, inhibitory control, and cognitive flexibility) on the NIH Toolbox battery (see Hessl et al. [38]). The lack of association could indicate inadequate validity of the BRIEF for this population or discrepancy between laboratory-based assessment and real-life EF. Therefore, further psychometric work on the application of the BRIEF to FXS and other ID groups is important prior to recommending its use in clinical trials.
4. Language measures.
  - Standardized tests. There are numerous standardized language tests that have proven useful in describing the profile of impairments characteristic of individuals with FXS [66]. These include the Peabody Picture Vocabulary Test-Fourth Edition (PPVT-4; [67]), for measuring receptive vocabulary, and the co-normed Expressive Vocabulary Test-Second Edition (EVT-2; [68]). The PPVT-4 and EVT-2 are direct, individually administered measures that are suitable for a wide range of age and ability. They provide growth scale values based on Rasch scaling, which would be useful for tracking intra-individual change. Other standardized tests that have been used to characterize language function in individuals with FXS include the Clinical Evaluation of Language Fundamentals-Fifth Edition (CELF-5) and CELF Preschool-Second Edition (CELF-P2) [69, 70], which have subtests for various language components, including expressive syntax, a problem area for individuals with FXS [66]. Despite their wide application in FXS and ID in general, none of these standardized tests have been formally evaluated in these populations in terms of psychometric properties of relevance for clinical trials. Pending more research, these standardized language tests could serve as *longer*-*term* outcome measures as they tap into more crystallized type of knowledge. Evaluation of measurement properties places them in the *no evidence available* category.
  - Fast-mapping measures. In contrast to standardized language tests, which largely measure the accumulated products of language learning, there have been recent attempts to create measures that index language learning in real time, an approach that is especially promising for *shorter*-*term* clinical trials. The best studied of these “process” measures for FXS are fast-mapping tasks. Fast-mapping is an associative learning process in which children form an initial representation of a word’s meaning by inferring a correspondence between a novel label and the speaker’s intended referent [71]. Fast-mapping tasks usually are limited to the earliest phases of learning a word. Several recent studies have demonstrated that fast-mapping tasks can be completed by minimally verbal males with FXS, as young as age 4 years [72]. These tasks also document age-related deficits in learning that distinguish males with FXS from similarly aged, cognitively matched males with nonsyndromic ASD [72–74]. Although most fast-mapping tasks are administered by an examiner in a face-to-face setting, computer-administered versions have been developed [75], increasing the feasibility of the task for clinical trials. Evidence of validity has been provided by studies documenting correlation with standardized test of vocabulary, such as the PPVT-4 [72–74]. Despite this, the type of psychometric studies needed to establish the appropriateness of fast-mapping tasks for clinical trials for FXS has yet to done, including evaluations of test-retest reliability and sensitivity to change. Thus, in terms of measurement properties, fast-mapping measures fall into the *no evidence available* category.
  - Expressive language sampling (ELS). In contrast to the forgoing measures, psychometric work has been conducted on ELS and the Working Group concluded that these procedures held the most promise for immediate use in clinical trials, provided that the samples are collected in contexts that are sufficiently structured to ensure consistency of the interaction across participants and occasions of measurement. Dyadic conversation with an examiner and narrative retelling have been the most widely used ELS procedures with individuals with FXS [76]. A pilot validation of ELS in FXS, including 36 males and females, aged 5–36 years, showed very good reproducibility and clinical validity and led to its use in three clinical trials [20]. Results of ELS assessments from these clinical trials have not yet been reported. A NICHD R01 project (HD074346; PIs: Abbeduto, Berry-Kravis, Sherman, Edgin, Sterling) was funded to perform optimization and validation of the ELS in FXS and Down syndrome across five sites and also to measure its response to change after 2 years. Pending the results of this work, we can conclude that ELS procedures could be used as either *shorter*- or *longer*-*term* outcome measures. Existing evidence supports a *moderate* tool quality label, although it should be noted that transcription of samples can be highly time-consuming which may make it difficult to use in very large trials unless increased automation of transcription can be developed or sample length can be reduced without loss of validity.
5. Social cognition. The Working Group stated that measures of social cognition have not been well studied in FXS. It was noted that the relationship between performance on such measures and actual social behavior is not strong [77]. The Working Group concluded that both the Social Responsiveness Scale (SRS) [78] and the use of eye-tracking technology to assess attention to social events were promising but required further validation in FXS.
  - Social Responsiveness Scale (SRS). This is a 65-item parent/caregiver/teacher rating scale used to assess children on five subscales: Social Awareness, Social Cognition, Social Communication, Social Motivation, and Autistic Mannerisms (Restricted Interests and Repetitive Behavior in SRS-2) [79, 80]. The second edition of the SRS (SRS-2; [78]) also contains a Preschool Form. The SRS-2 can be used as a screener in clinical or educational settings, an aid to clinical diagnosis, or a measure of response to intervention, but questions remain about its usefulness as an outcome measure sensitive to change in clinical trials in part because the scale’s criterion validity is lower than the ADI-R’s [15]. The SRS’s psychometric properties are rather robust. Factor analyses support a one-factor solution in populations at risk for ASD, limiting the SRS’s use to an overall assessment of social responsiveness [78]. The SRS’s internal consistency [80–82] and test-retest reliability [81–83], including parents-teachers [83, 84] and mother-father [81] correlations, indicate reasonable psychometric properties (e.g., good to excellent test-retest reliability). In addition, published data support good convergent and discriminant validity of the SRS with the Child Behavior Checklist (CBCL) [81, 82]. The SRS seems suited for studies evaluating *shorter*- or *longer*-*term* effects of interventions. Because the SRS has only been applied to a single open-label trial in FXS, we labeled it as a *limited*-*moderate* tool. Additional information is provided in the section “Autistic behavior”, under the “Behavior and Emotion measures” section.
  - Eye tracking/pupillometry. Eye-gaze avoidance is a major feature of FXS [2, 3]. A pilot study of social gaze behavior in FXS quantified gaze avoidance and pupillary reactivity to emotional faces, including 15 males and females, aged 7–51 years, revealing good feasibility and excellent test-retest reliability [85]. A customized eye-tracking configuration was used to quantify social gaze while engaging in a naturalistic face-to-face social interaction with a female experimenter as opposed to static faces used in prior protocols [77]. Participants with FXS spent significantly less time looking at the examiner’s face and had shorter episodes of social gaze than controls. It was proposed that this paradigm could be employed in clinical trials to provide a more naturalistic measure of social gaze than protocols using images of faces [77, 86]. To date, the abovementioned eye-tracking protocol [85] has been used in several clinical trials, with preliminary evidence of pupillometry’s sensitivity to treatment in a minocycline trial [87] as well as improvement in social gaze in a mavoglurant trial [88]. Data from other clinical trials are awaiting analysis for evidence of sensitivity to change. Then, eye tracking/pupillometry in FXS could be used as a *shorter*-*term* outcome measure with evidence for *moderate* to *strong* tool quality. Although eye-tracking protocols can be easily standardized across sites, one potential limitation is the cost of eye-tracking equipment that may limit its utility in smaller trials.
  - Self-Perception Profile for Adolescents (SPPA). The application of the SPPA, which elicits self-ratings of social relationships, friendships, and self-worth, among other domains, in recent social cognition studies revealed significant differences between adolescent females with FXS and typical age-matched peers [89]. Moreover, scores on the SPPA correlate with other social-cognitive measures, such as “reading” thoughts and feelings from a photograph of a face [90]. Parent reports and the SPPA were discrepant, with parents rating adolescents with FXS as more impaired than the adolescents rated themselves, which is a common finding among several populations. Awareness of the discrepancy between self and parent perceptions of social functioning is important for determining the need for intervention and its assessment. At present, although a promising measure, evidence for the use of the SPPA in clinical trials is lacking (i.e., *no evidence available* category).
6. Academic achievement. The Working Group recommended the use of academic achievement measures as indicators of *longer*-*term* outcomes. The expectation is that changes in language, memory, and EF are likely to lead to changes in the academic domain. In order to better understand the academic domain in FXS, language skills, reading ability, and phonological awareness have been studied in affected boys. As in typically developing boys, reading skills are also significantly correlated with delayed phonological awareness and level of non-verbal cognition in boys with FXS [90]. Young males with FXS display reading abilities that are commensurate with their cognitive expectations; however, their phonological skills are weaker than expected [91]. In conclusion, evidence is lacking on academic achievement and related measures for determining their usefulness and tool quality in FXS clinical trials (i.e., *no evidence available* category).
7. Learning process measures. Process measures may also be more sensitive to *shorter*-*term* drug effects than “product” measures, which are essentially the result of a learning process and, therefore, “accumulate” gradually to measurable levels of relevance to daily functioning and quality of life [92]. For example, in a recent study employing a “process” measure of change, Hall and colleagues [93] examined the rate at which lower-functioning boys with FXS, aged 10–23 years, were able to learn the relationships between mathematical stimuli after 2 days of training presented either on a computer or by a therapist [92]. By tracking learning on a trial-by-trial basis, these authors found that boys with FXS were significantly slower to learn to match fractions to pie charts in comparison to age- and IQ-matched controls with ID [92]. These data suggest that meaningful differences in the rate at which boys with FXS learn new material could be detected in this population. Studies expanding on these findings are also needed to determine the usefulness of “process” measures for clinical trials in FXS.

### Potential measures

1. Learning paradigms. This type of measures, with dynamic and regular training over time, may have excellent potential for use as cognitive endpoints in FXS clinical trials. In contrast to traditional outcome measures, which are evaluated at several key points during a trial, learning paradigms can provide data on progress much more often, providing a detailed “slope” of progress or change. Moreover, learning paradigms might more closely match progress in academic achievement or acquisition of skills as they occur in the individual’s environment rather than laboratory-based measures. In a sense, learning paradigms reflect a non-pharmacological intervention, which may progress more rapidly in clinical trials due to synergistic effects with a targeted pharmacological treatment. The ongoing Cogmed Working Memory Training FXS clinical trial (PI: Hessl), supported by the John Merck Fund, provides an example of how daily cognitive training can be tracked on a trial-by-trial basis, yielding a high-resolution slope of change. Results of this double-blind placebo-controlled study should be available in 2017; however, open-label results of a FXS Cogmed feasibility study involving 17 males and females, mean age 12.49 years, were reported by Au and colleagues [94].
  Learning paradigms focused on language have recently been shown to have efficacy for individuals with FXS over a wide age range (e.g., [95, 96]) and, like Cogmed, could be useful as outcome measures, with the added benefit of “boosting” the effects of pharmacological treatments.
2. Novel adaptive behavior measures.
  - Independent Living Scales (ILS; [97]). The ILS is a measure of adaptive behavior that assesses the ability to function independently and handle real-life situation. It was originally designed to evaluate adaptive behavior in aging populations but has recently been employed to evaluate adaptive skills in individuals with FXS [98]. The ILS has five subdomains, two composite factors, and a full-scale standard score ranging from 55 to 115. Psychometric properties, such as test-retest stability and internal consistency, have been reported to be strong [97] but they have not been investigated in detail in FXS. In a recent study mentioned above [99], 70 individuals with FXS (males and females), aged 15–25 years, showed comparable ILS domains and factors to those of age/IQ-matched individuals with ID [98]. The authors used raw scores to conduct the comparison because 77% of males and 17% of females with FXS were found to have scores at a floor level for the test [98]. Therefore, the ILS appears to have *limited* utility as a directly administered *longer*-*term* outcome measure of adaptive behavior for males with FXS, and another recent study shows that it is also promising for use in affected females [99].
  - Waisman Activities of Daily Living (W-ADL) Scale. Another measure with potential for adolescents and adults with developmental disabilities is the W-ADL, which has been recently tested in 1014 individuals including 147 with FXS [100]. The W-ADL covers 17 daily living activities, and it has shown excellent psychometric properties, including internal consistency, criterion and construct validity (e.g., correlated with Vineland scales screener), and no floor or ceiling effects. Importantly, W-ADL scores differentiate maternally reported level of ID (mild, moderate, severe, profound) [100]. A recent application of the W-ADL to 147 adolescents and adults with FXS, aged 12–48 years, showed an improvement in adaptive skills over time [101]. While the W-ADL is recommended for surveys and epidemiological research, it may also be useful as an outcome measure for clinical trials.
  - Scales of Independent Behaviors-Revised (SIB-R; [102]). While only applied as a measure of maladaptive behavior, the SIB-R is mainly an adaptive behavior instrument that has not been evaluated systematically in FXS.

### Conclusions

Most of the conclusions of the Working Groups continue to be valid. As the 2013 Report indicated, there is only sparse evidence on reliability and validity for most of the instruments used to measure cognitive deficits in FXS. However, several pilot projects with high potential cognitive measures (KiTAP, ELS, NIH-TCB) have been completed since the 2013 Report and larger validation studies are in preparation or ongoing. Thus, new data in the Cognition domain may lead to stronger recommendations for some tools in the next few years. In terms of adaptive behavior, an area with a long track record of observational studies in FXS, new measures including the ILS, and the W-ADL, are promising but need to be formally assessed. It is expected that the recently released Vineland-3 will perform similarly to the Vineland-II in FXS, although will be more relevant to current adaptive skills (e.g., electronic device use). Studies to evaluate the Vineland-3 in FXS are underway. We conclude that the overall evidence for cognition-related outcome measures in FXS places them in the *limited* to *moderate* quality range, with most instruments adequate for assessment of *longer*-*term* changes (i.e., some evaluating specific cognitive functions may be appropriate as *shorter-term* endpoints).

## Behavior and Emotion measures

This section covers some of the most distinctive phenotypical features of FXS, which are a major focus of current clinical management [2]. The range of behavioral abnormalities in FXS is wide, with five areas commonly recognized: maladaptive/disruptive behaviors, ADHD-like behaviors, stereotypic and repetitive behaviors, anxiety, and autistic features [3]. In addition to the frequent combination of anxiety and autistic features with other problematic behaviors, hypersensitivity or over-reactivity (hyperarousal) to stimuli complicates the delineation of abnormal behaviors in children with FXS [3]. Other general issues that deserve consideration include the fact that (1) most behavioral measures have been developed for populations with relatively normal cognitive function, (2) without the specific goal of detecting change over time, and that (3) the conceptual framework/construct underlying specific abnormal behaviors is not fully developed in FXS, and (4) may differ from the one applied to the general population (e.g., anxiety in intellectual disabled groups such as FXS). The latter points have also been raised in the reviews of outcome measures in ASD [10–15]. Here, we review measures of abnormal Behavior and Emotion divided into the aforementioned five areas.

In 2013, the Working Group on Behavior and Emotion made the following general recommendations regarding outcome measures targeting the behavioral domain: (1) to determine the psychometric properties, namely reliability, validity, and sensitivity (including sensitivity to change), of several currently available behavioral measures within the FXS population; (2) to establish the specificity of the measured constructs to FXS by examination of correlations between such measures and valid FXS biomarkers (e.g., brain imaging) and gene-dose (e.g., FMRP); (3) to consider the development of a new behavior rating scale for FXS to cover the phenotype and the full range of associated symptoms; and (4) to supplement traditional psychometric studies with data from focus groups that include patients or their proxies and other caregivers to provide input on the construct validity, interpretability, and feasibility of measures of interest. We will review these general recommendations for the Behavior/Emotion domain in each specific section (subdomain).

Table 3 depicts behavioral and emotion outcome measures, their suitability for quantifying *shorter*- vs. *longer*-*term* effects in clinical trials and the quality of their measurement properties. In general terms, because of the dynamic nature of problematic behaviors, their measures tend to reflect *shorter-term* effects. However, because many instruments can be applied repeatedly without a training effect, behavioral endpoints could also be used for evaluating *longer*-*term* outcomes. The field is dominated by informant-based rating scales using Likert type of scoring, with variable quality of measurement properties. An additional discussion of these issues is provided in the following “Problem behaviors: focus on disruptive behavior domain” section.

**Table 3:** Outcome measures of problem behaviors: focus on disruptive behaviors domain in fragile X syndrome

﻿Subdomain	Outcome measure	*Shorter term*	*Longer term*	Quality of tools
Irritability/aggression				
	ABC-C_I	Yes	Yes	+++ [54, 55, 104–110]
	ABC-Cfx_I	Yes	Yes	+++ [9, 46, 103, 111–113]
Stereotype/self-injury/aggression	BPI-S	Yes		+ [115–117]
Variety of behaviors	FXS Rating Scale	Yes	Yes	+ [63, 114]
Inattention	Conners/ADHD-RS	Yes		++ [44, 64, 120, 122, 126]
Hyperactivity/impulsivity	Conners/ADHD-RS	Yes		++ [44, 64, 120, 122, 126]
Hyperactivity/impulsivity/inattention	CBCL_ADHD Scale	Yes		++ [130, 131]
Hyperactivity/impulsivity/inattention^a^	SNAP-IV	Yes		++ [40, 113, 267]
Anxiety				
Social withdrawal/anxiety	ABC-C_SW	Yes	Yes	+++ [9, 19, 28, 46, 103, 111–113]
Social anxiety				
	PARS	Yes	Yes	++ [134, 138]
	ADAMS	Yes	Yes	++ [13, 17, 113, 132, 138]
	ADAMS Social Avoidance	Yes	Yes	++ [17, 139]
	CBCL Withdrawn	Yes	Yes	+ [18, 19, 135]
	CBCL’s DSM-Anxiety	Yes		+ [136, 137]
	SCARED	Yes		+ [10, 13]
Repulsive/compulsive behavior	RBS-R	Yes		+ [15, 179–184]
Social reciprocity/avoidance	SRS	Yes	Yes	+/++ [15, 46, 78–81, 112, 113]

*Abbreviations*: *ABC-C* Aberrant Behavior Checklist-Community Edition, *ABC-C_I* ABC-C, Irritability subscale, *ABC-C*_*FX_*_ *I* ABC-C_,_ FXS-specific, Irritability subscale, *BPI* Behavior Problems Inventory, *ADHD-RS* Attention-Deficit Hyperactivity Rating Scale, *CBCL_ADHD Scale* Child Behavior Checklist_ADHD subscale, *SNAP-IV* Swanson, Nolan and Pelham Questionnaire-4th ed. *ABC-C_SW* ABC-C, Lethargy/Social Withdrawal subscale, *PARS* Pediatric Anxiety Rating Scale, *ADAMS* Anxiety Depression and Mood Scale, *CBCL Withdrawn* CBCL Withdrawn Subscale, *CBCL’s DSM-Anxiety* CBCL’s Diagnostic and Statistical Manual-Anxiety Subscale, *SCARED* Screen For Child Anxiety Related Emotional Disorders, *RBS-R* Repetitive Behavior Scale-Revised, *SRS* Social Responsive Scale, *shorter term* deemed suitable for clinical trials lasting <12 months; *longer term* deemed suitable for clinical trials lasting >=12 months. Grading system: *+++* strong, *++* moderate, *+* limited evidence, *?* unknown/poor methodological quality, *blank cell* no evidence available

^a^Oppositional and defiant items are also included

### Problem behaviors: focus on disruptive behavior domain

#### Background

Maladaptive behaviors, in particular externalizing behaviors, termed here “problem behaviors”, are one of the major clinical concerns with functional and quality-of-life implications in FXS [3, 103]. Therefore, there is a greater experience in this behavioral area than in others. Measures previously used in clinical trials in FXS cover a wide range of problem behaviors, including ADHD-like behaviors (e.g., hyperactivity), stereotypic and repetitive behaviors, and anxiety. Because of the large volume of information on each of these types of behaviors, they will be reviewed in separate sections (i.e., subdomains). In this initial section, we will focus on irritability/agitation/aggressive behavior, termed here disruptive behavior, and self-injurious behavior. As stated in the 2013 Report, problem behaviors are typically evaluated by informant-based rating scales, which are completed by a parent, caregiver, or clinician. We continue to consider this a reasonable approach because of their ease of use, cost, and application to multi-site studies. However, it is important to point out that these measures involve evaluations of behavioral abnormalities during the last few weeks prior to rating and represent average estimates of a behavioral “style” rather than a dynamic process. By far, the best characterized instrument is the Aberrant Behavior Checklist-Community (ABC-C) [104]. The ABC-C covers five categories of problem behaviors, but it is limited in its evaluation of ADHD-, anxiety-, and autistic-like behaviors. As depicted in Table 3, the ABC-C has been widely applied to trials involving individuals with ID or ASD [104–106]. The 2013 Report summarized several limitations of the ABC-C (e.g., test-retest reliability) that can affect its sensitivity to change and, therefore, its ability to detect response to treatment. Despite this, the ABC-C has generally good psychometric properties and a successful track record for documenting improvements in disruptive behavior in controlled trials of idiopathic ASD [107, 108] and open-label trials of lithium, aripiprazole, and donepezil in children and adults with FXS [55, 109, 110] (Table 3). As other measures that were developed for ID or ASD, the relevance of the ABC-C to the FXS behavioral phenotype characterized by prominent anxiety-like behaviors was questioned. Therefore, the Working Group decided to revise and validate the ABC-C for FXS and, after improving its psychometric properties, to determine its sensitivity in clinical trials.

#### Progress and plans in FXS

1. Aberrant Behavior Checklist-Community (ABC-C), adapted for FXS (ABC-C_FX_). The process of refactoring the ABC-C for FXS, through a multi-site Fragile X Clinical and Research Consortium (FXCRC) collaboration [111], led to the elimination of three items and, more importantly, to a different factor structure that added novel subscales that seem to better represent autistic and social anxiety behaviors. The ABC-C_FX_ factor structure was further supported by a subsequent study conducted by Novartis and RTI/Our Fragile X World [103]. The ABC-C_FX_ has been used in every drug trial performed in FXS since its publication in late 2011, including a pediatric arbaclofen trial that showed some significant improvements [112, 113]. Based on these controlled trials and their open-label extensions, it has been concluded that the ABC-C_FX_ could be used as either a *shorter*- or *longer*-*term* outcome measure (see Table 3). Considering its initial psychometric evaluation [111], re-assessment [103], and application to FXS trials, in which several subscales have demonstrated sensitivity, the ABC-C_FX_ falls into the *moderate*-*strong* category of instruments (two positive randomized placebo-controlled trials: phase 2 mavoglurant [9], phase 3 pediatric arbaclofen [46, 113]). This labeling corresponds mainly to the ABC-C_FX_ Irritability subscale, pertinent to the Disruptive Behavior domain. However, two other ABC-C_FX_ subscales, specifically Social Avoidance and Hyperactivity, demonstrate many of the tool quality features of the Irritability subscale in a phase 3 pediatric arbaclofen study [113]. The main criticism continues to be its limited coverage of some key areas (e.g., non-social anxiety), which is discussed below, as well as the susceptibility to placebo effects. Nonetheless, rating variability and placebo effects are not specific to the ABC-C_FX_ but are associated with any parent/caregiver report measure.
2. Fragile X Syndrome Rating Scale (FXSRS). As concluded by the Working Group, development of a new behavior rating scale covering the FXS behavioral phenotype and associated symptoms is a worthwhile effort. An initial attempt has been completed in the context of a trofinetide phase II trial conducted in adolescents and adults with FXS by Neuren Pharmaceuticals [63, 114]. The FXSRS is a 34-item rating scale that covers a wide range of behavioral symptoms, including a FXS Core Phenotype subscale (i.e., 10 symptoms more prominent or prevalent in FXS than in other neurodevelopmental disorders), a FXS with Autism Phenotype subscale (i.e., six features shared to a similar degree by FXS and idiopathic ASD), and an Associated Phenotypic Features subscale (i.e., 18 features shared to a similar degree by FXS and general ID) subscales [114]. Items are answered using a 4-point severity or frequency scoring system. Publication of the FXSRS is pending; however, description of features in a group of 70 males with FXS, aged 12–45 years, presented at a meeting in 2014 [114], suggests it meets the requirements stated in the 2013 Report, including its potential use as *shorter*- and *longer*-*term* outcome measure (Table 3). Although grading of the FXSRS at this point is not possible or *limited*, it was employed in the aforementioned trofinetide study. This trial was reported as positive, based on an analytical strategy using multiple endpoints including the FXSRS (Berry-Kravis et al. 2016; www.neurenpharma.com/IRM/PDF/1557/TrofinetidesuccessfulinPhase2trialinFragileX).
3. Behavior Problems Inventory-Short Form (BPI-S; [115]). The BPI-S is a 30-item parent report measure that includes aggressive/destructive, self-injury, and stereotypic behaviors subscales rated over the previous 2-month period. Items are answered using a 5-point frequency and a 4-point severity scoring system. Studies employing an earlier version of the scale (BPI-01; [116, 117]) reported that in 50 males with FXS, aged 8–24 years, 79% engaged in self-injurious behavior (SIB), 98% in stereotypic behavior, and 75% in aggressive/destructive behavior. Moreover, 33% of the sample demonstrated aggression and 80% stereotypic behavior, both on a daily basis. The BPI-S could be used as either *shorter*- or *longer-term* outcome measure. However, it has not been subjected to reproducibility analyses in FXS and, at present, it is a *limited* quality tool.

#### Potential measures

A review of the ID and ASD literature supports the need for behavioral measures with better psychometric properties, particularly those relevant to detecting response to interventions. The recent National Institute for Health Research’s review of behavioral outcome measures in ASD, mentioned in the “Material and methods” [11], concluded that there was “patchy evidence on reliability and validity”. Of the six measures under evaluation, only two had acceptable properties [12]: the Child Behavior Checklist (CBCL) and the Home Situations Questionnaire-Pervasive Developmental Disorders version (HSQ-PDD). As also suggested by observational studies in FXS [18, 19], CBCL’s main limitation is the lack of evidence on content validity for use with individuals with ID or ASD, particularly for those below the mild ID level. On the other hand, the HSQ-PDD has potentially relevant items but it is a relatively newer measure still under development. Thus, the CBCL and HSQ-PDD are instruments with potential as supportive outcome measures, but not as primary endpoints in intervention studies in FXS.

#### Conclusions

The ABC-C_FX_ is an adequate instrument for disruptive behavior in FXS, with measurement properties at the *moderate*-*strong* quality level, with the ABC-C_FX_ Irritability subscale demonstrating sensitivity in two FXS trials [112, 113] which could be used as either a *shorter*- or *longer*-*term* outcome measure. Despite the strengths of the ABC-C_FX_, the fact that some key problem behaviors are not represented and that many items are not disorder-specific, the recommendation in the 2013 Report to develop a more comprehensive and FXS-specific outcome measure is still valid. The FXSRS could meet this apparent need; however, data are not yet available for a complete evaluation of this novel instrument.

### Attention-deficit hyperactivity disorder (ADHD)-like behaviors

#### Background

Characterized by hyperactivity-impulsivity and/or inattention symptoms (DSM-5) [39], ADHD is one of the most common and potentially impairing behavioral issues in individuals with FXS. Evidence of its importance comes from family surveys [103, 118], several observational studies (reviewed in [3]), and a recent clinical study reporting that school-age children with FXS were significantly more likely to receive a professional diagnosis of ADHD compared to similar aged children with either Prader-Willi syndrome, Williams syndrome, or velocardiofacial syndrome [119].

#### Progress and plans in FXS

A limited number of recent clinical trials in FXS have included measures of ADHD as primary or secondary endpoints (Table 1), in addition to the ABC-C_FX_ (see the “Problem behaviors: focus on disruptive behavior domain” section) that, as mentioned in the preceding section, includes a 16-item Hyperactivity subscale with many of the features of the strong Irritability subscale [103, 111].

ADHD Rating Scale-IV (ADHD-RS-IV; [120]). In the previously mentioned randomized phase II trial of metadoxine in FXS (AL014 trial), Berry-Kravis and colleagues (2015) found no significant reduction over placebo in scores on the Inattentive subscale of ADHD-RS-IV [120] and no differences between drug and placebo on the total score of the ADHD-RS-IV (employed as a secondary outcome measure) [44]. The ADHD-RS-IV contains 18 items that correspond directly to the symptoms of ADHD [120] on the Diagnostic and Statistical Manual for Mental Disorders-4th Edition (DSM-IV) [121] and the most recent DSM-5 version [39]. A clinician-administered version of the scale is also available [122], which was used with some adaptations for ID in the MDX trial [122]. Although the psychometric properties of the ADHD-RS-IV have been reported to be good, there are no normative data available for this scale in individuals with FXS, and many of the items are difficult to interpret in FXS due to confounding ID. The scale could be used for measuring *shorter*-*term* effects, but it has *limited* tool quality due to the problems with content and interpretation in FXS.

#### Potential measures

Four potential measures of ADHD that have been employed in recent studies in FXS [123–126] are summarized below.

1. The ADHD Test (ADHDT; [123]). A study employing the ADHDT reported that 68 males and females with FXS, aged 15–25 years, displayed a good range of scores [125]. The ADHDT contains 36 items divided into 3 subscales: Hyperactivity (13 items), Impulsivity (10 items), and Inattention (12 items). The ADHDT was normed to a group of 3- to 23-year-old individuals who met the DSM-IV criteria for ADHD. The psychometric properties of the ADHDT have been reported to be good, but its only application to FXS is as a measure of ADHD symptoms in conjunction with the ABC-C_FX_ Hyperactivity subscale.
2. Conners Rating Scales-Revised [124]. Another recent study involving 46 boys with FXS, aged 4–11 years, showed “stable and striking impairments in inattention” on the Conners Teacher Rating Scale (CTRS) and the Conners Parent Rating Scale (CPRS) [126]. Both scales have four subscales: ADHD Index, Hyperactivity, Cognitive, and Oppositional, scored on a 4-point Likert scale, and an age-normed *T* score above 70 on the ADHD index is indicative of an ADHD diagnosis. The CPRS has reported good to excellent internal consistency across the four scales [124, 126], with scores on the CPRS generally higher than on the CTRS.
3. Child Behavior Checklist (CBCL)’s ADHD subscale [127]. Several observational studies have employed the CBCL (ages 1.5–5 years, ages 6–18 years; [127, 128]) as a measure of ADHD symptoms in FXS [reviewed in [3]. The CBCL is a 118-item parent rating scale used to assess internalizing and externalizing symptoms in children without ID. Items are answered using a 3-point Likert scale. The DSM-ADHD scale is one of several DSM scales of the CBCL based on criteria from the DSM-IV, with *T* scores above 70 being indicative of an ADHD diagnosis. The CBCL was normed on a large, representative sample of children and adolescents and, in this population as well as in idiopathic ASD, has overall good psychometric properties [11, 129]. In a recent longitudinal study employing this measure, Grefer and colleagues reported that the mean DSM-ADHD raw scores increased over a 2-year period in a sample of 33 preschool-aged boys with FXS [130]. The percentage of boys who obtained *T* scores in the clinical range also increased from 9 to 12% over the same period. However, considering that the norms of the CBCL were not developed for children with ID, the reliability of this scale for individuals with FXS is unknown, and it is unclear whether this scale can be useful in clinical trials given its narrow range of scores.
4. The SNAP-IV [40]. This is a revision of the Swanson, Nolan and Pelham (SNAP) Questionnaire [131]. The SNAP-IV is an 18-item questionnaire addressing symptoms of ADHD (72 additional items cover ADHD-related behaviors such as those of Oppositional Defiant Disorder). It has been used in a completed (ampakine CX516) study [40] and an ongoing (ganaxolone) trial in FXS, showing very good test-retest reproducibility but no sensitivity to change in the CX516 trial [40].
  Overall, all these potential instruments for evaluating ADHD symptoms in FXS are promising *shorter*-*term* outcome measures, but their tool quality fall in the *limited* to *no evidence available* quality range.

#### Conclusions

Available ADHD rating scales seem particularly suited to studies evaluating *shorter-term* effects in FXS (Table 3); their measurement properties are at the *limited* to *moderate* tool quality range (Table 3). This evaluation may change after ADHD measures are fully evaluated in intervention studies in FXS.

### Anxiety

#### Background

Anxiety-like behaviors in FXS are prevalent and frequently severe [3, 132]. Two features distinguish anxiety in FXS; its prominent social component and close relationship with and difficult differentiation from hyperarousal. Until recently, most data on anxiety in FXS was based on informal observations. The ABC-C has been the only standardized measure employed in a systematic way to characterize social anxiety, usually in the context of a general assessment of problem behaviors. As for other disorders associated with ID, delineating and quantifying anxiety in FXS is challenging because of the limited behavioral repertoire of individuals with cognitive impairment and their inability to report emotional states. Interestingly, in FXS anxiety-like behaviors seem to be stable across the age spectrum [103]. This suggests that, as in the general population, anxiety-like behaviors tend to be less dynamic than other problem behaviors and, consequently, instruments measuring them may be able to detect either *shorter*- or *longer*-*term* changes. Most standardized data on social anxiety in FXS has been collected using the ABC-C, which in its original form includes a subscale covering a variety of abnormal social interaction behaviors (i.e., ABC-C Lethargy/Social Withdrawal) [104]. Among this subscale’s items, there are some corresponding to social anxiety. These were initially characterized as such by observation [19, 28] and, more recently, by factor analysis that generated the new Social Avoidance subscale of the FXS-specific ABC-C_FX_ [111]. Although the overall psychometric properties of the ABC-C_FX_ are good to excellent [103, 111], the fact that the Social Avoidance subscale includes only four items may limit its sensitivity (see Berry-Kravis et al., in press) [113]. This concern is also extended to the ABC-C_FX_ Inappropriate Speech subscale. Nonetheless, the ABC-C_FX_ Social Avoidance did show sensitivity to treatment in a randomized placebo-controlled phase 2 arbaclofen trial [112]. Since items directed at other forms of anxiety are not included in the ABC-C_FX_, different measures have been explored and considered potentially suitable but their sensitivity is still unknown. The Anxiety, Depression and Mood Scale (ADAMS) [133] was developed as a tool for screening anxiety and mood symptoms in individuals with ID (Table 3). The ADAMS has shown convergent validity with a caregiver diagnostic interview for anxiety in FXS [132] and psychometric evaluations in adults with ID revealed adequate internal consistency and convergent and discriminant validity [115, 133]. Nonetheless, ADAMS’s sensitivity to interventions is unknown. The Working Group also identified the Pediatric Anxiety Rating Scale (PARS) [134] as a promising measure, based on its successful application to selective serotonin reuptake inhibitor trials in pediatric anxiety disorders (Table 3). Other measures employed for measuring anxiety-like behaviors in FXS include the CBCL’s Withdrawn subscale [19, 135] and CBCL’s DSM-Anxiety subscale [136, 137]. Psychometric properties of these CBCL subscales in FXS, including their sensitivity to change, are unknown. Nonetheless, because of its content, more adequate for individuals in the normal or borderline IQ range, and limited range of scores, the CBCL’s use in FXS clinical trials may be limited.

#### Progress and plans in FXS

1. Pediatric Anxiety Rating Scale (PARS). A pilot observational study of the PARS in FXS, involving 49 subjects, aged 5–35 years, confirmed its potential for clinical trials. It demonstrated good psychometric properties, including feasibility, reliability, and convergent validity [138]. Responsiveness to change (i.e., sensitivity) is still unknown, although this information may become available soon since the PARS has been already implemented in three clinical trials.
2. The Anxiety, Depression and Mood Scale (ADAMS). A recent observational study of the ADAMS in Rett syndrome concluded that, among measures of anxiety-like behaviors, it has the best psychometric properties [17]. Although sensitivity was not examined in this investigation, it included an assessment of the ADAMS’ functional relevance. ADAMS scores correlated inversely with adaptive behavior skills and quality of life scores [17] indicating that this is a clinically meaningful measure for interventions (as defined by the FDA) for Rett syndrome and probably other neurodevelopmental disorders such as FXS. Moreover, a pilot open-label trial with mecasermin (recombinant human IGF-1) in Rett syndrome demonstrated that the ADAMS Social Avoidance subscale’s was mildly sensitive to response to treatment [139]. A recently completed but not yet reported trial of ganaxolone in FXS employed, in addition to the PARS, the ADAMS.

#### Potential measures

As mentioned in the “Problem behaviors: focus on disruptive domain” section, review of the ID and ASD literature supports the need for anxiety measures with better psychometric properties, including greater sensitivity to interventions. The comprehensive examination of tools to measure outcomes in young children with ASD, conducted by the UK’s NHS National Institute for Health Research, also covered anxiety [10]. The review found literature on eight measures for high-functioning children with ASD. Of these questionnaires, only three had strong measurement properties: the Spence Children’s Anxiety Scale (SCAS), the Revised Children’s Anxiety and Depression Scale (RCADS; revised SCAS), and the Screen for Child Anxiety Related Emotional Disorders (SCARED). Despite this, no data are available on the sensitivity of these three measures to change or intervention [10]. A similar review of anxiety measures for clinical trials involving individuals with ASD, an effort sponsored by Autism Speaks, concluded that, of 10 reviewed instruments, only four were considered clinically relevant and adequate “with condition” [13]. This meant that, despite adequate reliability and validity, not all psychometric properties were present (e.g., limited information on test-retest reliability). The four measures were the Child and Adolescent Symptom Inventory-4th Edition Revised (CASI-4R), the Multidimensional Anxiety Scale for Children (MASC), the Anxiety Diagnostic Interview Scale for DSM-IV (ADIS), and the aforementioned PARS [13]. Two measures evaluated positively in the British review, namely the RCADS and the SCARED, as well as the ADAMS were determined by Lecavalier and colleagues to have potential for clinical trials [13]. Both reviews recommended the development of outcome measures with better properties for trials focused on anxiety in ASD. Although some of the measures identified in these reviews on ASD are adequate for individuals with FXS, the content of others is not compatible with a level of functioning on the moderate to severe range of ID.

#### Conclusions

Evidence for the PARS and ADAMS reviewed here support their promising status as *moderate* quality tools, in part because of the lack of information on sensitivity to change. As indicated above, these instruments could be used for measuring either *shorter*- or *longer*-*term* outcomes. Until these tools are fully evaluated and shown to detect change in intervention studies, it will not be clear if they are adequate as primary endpoints for the assessment of anxiety in FXS. A few other measures, such as the RCADS and the SCARED, could be potentially useful in higher functioning individuals with FXS (i.e., most females). Nevertheless, considering that anxiety is a major behavioral abnormality in FXS, development of anxiety measures with better psychometric properties is still a worthwhile goal.

### Autistic behavior

#### Background

In DSM-5, ASD is characterized by qualitative impairments in social interaction and communication, as well as restricted interests and repetitive behaviors (RRBs; [39]). RRBs are also prevalent in ID without ASD. Therefore, RRBs that are mainly linked to cognitive impairment are addressed in the following subsection. The inclusion of a number of explicit specifiers, among them cognitive and language abilities, is a major change to the definition of ASD under the DSM-5 criteria that affects both individuals with idiopathic ASD and those with FXS who show severe autistic behaviors. Establishing the diagnosis of ASD can be a challenge in FXS, as in ID in general, since demonstrating the selectivity of the social interaction impairments (i.e., beyond overall cognitive or language impairment) can be very difficult [2, 16]. This issue has been recognized in an expert consensus document on clinical practice from the Fragile X Clinical and Research Consortium [140] and a publication on ASD in FXS (see Kaufmann et al., in press) [141]. As in the general population with ASD [142, 143], severity of autistic behavior in FXS is variable and associated with age and IQ [18, 144]. Severity of autistic behavior tends to be stable [103] or improve [144, 145] over time. However, one study reported increase in autistic behavior with age, as measured with the Childhood Autism Rating Scale (CARS; [146]) that contrasted with most other studies using gold standard instruments (e.g., Autism Diagnostic Interview-Revised (ADI-R); [144]). Therefore, the specific instrument used in the assessment of autistic behavior may influence the outcome of studies in FXS.

#### Progress and plans in FXS

For the reasons stated above, the Working Group endorsed efforts at establishing the reliability, validity, and sensitivity to change of several available measures of autistic behavior in FXS. Most of this proposed work has not yet been carried out. Therefore, the following section reviews the potential of several relatively well-established measures of autistic behavior for evaluation of individuals with FXS. We also briefly discuss efforts at determining outcomes in idiopathic ASD that may also lead to improvements in autistic behavior assessment in FXS.

#### Potential measures

McConachie and colleagues provided the most comprehensive to date review aimed at identifying the quality of tools used for measuring outcomes for young children with ASD, a project of the UK’s NHS National Institute for Health Research [11]. A total of 17 measures of autistic behavior severity, 7 measures of social awareness, 4 tools for evaluating RRBs, and 2 instruments for specifically evaluating outcome of interventions, as well as instruments for other cognitive and behavioral domains, were included in this report. A parallel effort sponsored by the Autism Speaks foundation examined measures in terms of their adequacy for clinical trials in idiopathic ASD. Two of the publications resulting from the latter endeavor are pertinent to this section. The first evaluated 38 measures of social communication, concluding that only 6 were appropriate for use as outcomes [14]. The second focused on RRB; 24 measures were examined and 5 were recommended for use “with conditions” [15]. Although some of the instruments were included in more than one review, the number of assessed measures and the depth of their analyses preclude their discussion here. Nonetheless, these reviews are certainly the most important systematic work of relevance to autistic behavior in FXS published since the 2013 Report. For details, we refer the reader to the original publications.

Overall, the authors concluded that only sparse evidence exists on reliability and validity for only a few of the tools used in young children with ASD, probably the group of greater relevance to FXS. Since our literature search revealed that only in idiopathic ASD there has been a systematic review of the tools in terms of measurement properties, we focus on those instruments without having separate sections for ASD in FXS and idiopathic ASD.

Table 4 lists measures of autistic behavior severity and RRB other than autistic behaviors, including their suitability for *shorter*- vs. *longer*-*term* clinical trials and quality of measurement properties using the COSMIN grading criteria. We review these instruments here and in the next section.

**Table 4:** Outcome measures of autistic and non-autistic repetitive behaviors in fragile X syndrome

Domain	Outcome measure	*Shorter term*	*Longer term*	Quality of tools
Autistic behavior				
	ADOS	Yes	Yes	+ [147–155]
	SRS	Yes		+/++ [15, 46, 78–81, 112, 113]
	CARS	Yes		+ [160–166]
	SCQ	Yes		+ [162, 167, 173, 174]
	RBS-R	Yes		+ [15, 179–184]
Repetitive non-autistic behavior				
	RBS-R	Yes		+ [15, 66, 179, 180, 184, 191, 192, 194]
	RBQ	Yes		+ [189]
	BPI-S	Yes		+ [179, 195, 196]

*Abbreviations: ADI-R* Autism Diagnostic Interview-Revised, *ADOS* Autism Diagnostic Schedule, *SRS* Social Responsiveness Scale, *CARS* Children Autism Rating Scale, *SCQ* Social Communication Questionnaire, *RBS-R* Repetitive Behavior Scale Revised, *RBQ* Repetitive Behavior Questionnaire, *BPI-S* Behavior Problems Inventory, Short Form, *Shorter term* deemed suitable for clinical trials lasting <12 months, *Longer-term* deemed suitable for clinical trials lasting >=12 months, Grading system: *+++* strong evidence, *++* moderate evidence, *+* limited evidence, *?* unknown due to poor methodological quality, *blank cell* no evidence available

1. Autism Diagnostic Observation Schedule-Generic (ADOS-G). The ADOS-G is a widely used and well-established semi-structured, interactive instrument designed to assess aspects of social reciprocal interaction, communication, stereotyped behaviors, and restricted interests and play [147]. It is included in this review because the ADOS-G Severity Score is an overall measure of autistic behavior severity [148] and it has been administered pre- and post-treatment in the Early Start Denver Model (i.e., early intervention) randomized controlled trials in idiopathic ASD [149, 150]. There have been some recent refinements of the ADOS-G algorithm, such as the development of the ADOS-Calibrated Severity Score (ADOS-CSS) [148] that is independent of age, IQ, and language level [148, 151, 152]. The ADOS-G and, in particular, the ADOS-CSS seem to be suited for *shorter*-*term* trials [149, 150]. Together, based on data on reliability [147, 153–155] and validity [147, 154–158], the tool quality of the ADOS measures is in the *limited* to *moderate* range in idiopathic ASD. However, because of the lack of studies in individuals with the disorder, ADOS measures should be considered as having *limited* tool quality for FXS.
2. Social Responsiveness Scale (SRS). The features of the SRS were reviewed in the preceding section on “Social cognition”. A single open-label 10-week trial of acamprosate involving 12 subjects with FXS, aged 6–17 years, demonstrated a significant improvement in the scores of the SRS and several other cognitive and behavioral measures [159]. As noted in the “Social cognition” subsection under the “Cognitive measures” section, the SRS is suited for evaluating either *shorter*-*term* or *longer*-*term* effects, and its tool quality should be considered in the *limited*-*moderate* range in FXS.
3. Childhood Autism Rating Scale (CARS). This is a widely used 15-item observation and parent/caregiver interview that quantifies the severity of behaviors associated with ASD, with total scores ≥30 strongly suggesting the presence of the disorder [160, 161]. The CARS, Second edition (CARS-2) is a more recently published clinician-completed rating also used to determine ASD symptom severity [162]. The CARS-2 is suitable for evaluating *shorter*-*term* effects; its reliability [160, 163, 164], validity [165, 166], and lack of application to trials in FXS place it at the *limited* tool quality level.
4. Social Communication Questionnaire (SCQ; originally called the Autism Screening Questionnaire; [167]). The SCQ is a 40-item parents/caregivers rating scale based on the ADI-R [168] that screens for current core ASD behaviors and at age 4–5 years (lifetime). Language items not suitable for non-verbal children can be omitted; it is scored according to language level (maximum score 32 or 39), with higher scores indicating more severe symptoms. Five studies have used the SCQ to characterize autistic behavior and its severity in FXS [141, 169–172]. However, none has examined measurement properties including sensitivity. The SCQ is adequate for evaluating *shorter*-*term* outcomes. SCQ’s reliability [173, 174] and validity ([163]; reviewed in [12]) support the *limited* tool quality level.
5. Repetitive Behavior Scale-Revised (RBS-R). RRBs are a broad range of behaviors that are subdivided into two conceptual categories [175]: “lower-order” motor actions associated with lower developmental levels and characterized by repetition of movement (e.g., dyskinesias, stereotyped and repetitive manipulation of objects, and repetitive forms of self-injurious behavior (SIB)), and more complex or “higher-order” behaviors associated with higher cognitive abilities (e.g., object attachments, insistence on sameness, repetitive language, and circumscribed interests) [176, 177]. Both categories of behavior appear to be a function of an overall cognitive/behavioral rigidity/lack of flexibility [178]. Although no single RRB appears to be specific to ASD, an elevated pattern of RRB occurrence, co-occurrence, and severity characterizes the disorder [179]. The RBS-R is the most widely applied instrument for evaluating RRBs. The RBS-R is a 43-item parent/caregiver rating scale that was normed on individuals with ID and revised to capture some of the complex RRBs observed in ASD [179]. The items have been grouped into six subscales: Stereotyped, Self-injurious, Compulsive, Ritualistic, Sameness, and Restricted interests. The RBS-R was designed for use in populations with ASD or related neurodevelopmental disorders [179, 180]. RBS-R’s factor structure has been empirically evaluated in multiple studies [181–184]; recently, Bishop and colleagues concluded that a five-factor structure (Sensory Motor, Restricted Interests, Self-injury, Compulsive, and Ritualistic/Sameness and Sensory Motor behaviors) was the best solution for the RBS-R [183]. The scale’s psychometric properties are variable, ranging from poor (test-retest reliability) to strong (internal consistency) [180]. Thus, its overall quality as tool in idiopathic ASD should be considered *moderate*. The RBS-R was applied in a study that found different profiles of RRBs in young boys, aged 3–5 years, with either FXS and ASD or idiopathic ASD [185]. Both groups were similar with respect to lower-order (motoric) RRBs, but differed in terms of more complex forms that were less severe in FXS with ASD [186]. Furthermore, Wolff and colleagues found positive correlations between Self-injurious total scores on the RBS-R and number of SIB topographies and bilateral caudate nuclei volumes in the FXS group comorbid for ASD [187]. This study also reported that the profile of RRBs remains stable in FXS from preschool through at least the middle school years. This is in contradiction with the analyses of the ABC-C_FX_ Stereotypy, which demonstrates a decline in scores over time [103]. To date, the only FXS-specific factor analyses conducted on any measure of RRBs are those performed for the development of the ABC-C_FX_ *.* Although the RBS-R has been used in some FXS trials, information on its sensitivity to change is not yet available. Thus, the RBS-R seems suited for evaluating *shorter*-*term* and possibly also *long*-*term* effects. In the review of measures of RRBs for trials in idiopathic ASD by Scahill and colleagues, the authors concluded that the RBS-R, as well as the ABC-C Stereotypy subscale, Stereotyped Behavior Scale (SBS), and the Repetitive Behavior Questionnaire (RBQ) (see the next section), are *appropriate with conditions* [15]. Based on the latter and the lack of data in FXS, the RBS-R’s tool quality should be considered *limited*. However, because of the relevance of RRBs to the FXS behavioral phenotype, the RBS-R and similar instruments should be further studied in terms of suitability for clinical trials for this genetic disorder.

#### Conclusions

The measures of autistic behavior reviewed here seem adequate as *shorter*-*term* endpoints. Although they show *moderate*-*strong* measurement properties in individuals with idiopathic ASD, the evidence is *limited* in FXS and their sensitivity to change in this population is *unknown* to large extent because core autistic behaviors have not been the target of trials in FXS.

### Repetitive behaviors other than autistic behavior

#### Background

Numerous behaviors included in the RRB category are frequently observed in a wide range of neurodevelopmental and neuropsychiatric conditions, not only ASD [188–190]. In this section, we focus on instruments for RRBs pertinent to ID only, with emphasis on the FXS phenotype. Available information on the differences in RRBs between ID and idiopathic ASD suggest that they are mainly quantitative [179]. Limited evidence suggests that FXS is associated with increased risk for RRBs. Indeed, perseverative speech is a hallmark feature often problematic in boys with the disorder [66, 191, 192]. Other RRBs that are also elevated in FXS in general include hand flapping, body rocking, and SIB [193]. Different measures have been used to assess the severity of RRBs in FXS. They include specific instruments such as the RBS-R [179] and components of tests developed for measuring overall problem behaviors (ABC-C_FX_;[111]). The Working Group endorsed efforts at establishing the reliability, validity, and sensitivity of several currently available measures of RRBs *other than autistic* within the FXS population.

#### Progress and plans in FXS

1. Repetitive Behavior Scale-Revised (RBS-R). Although RRB categories have been reported in FXS at a global level, there is limited research characterizing the relative frequency of different types of RRBs or association with other factors. The RBS-R, discussed in more detail in the previous section on “Autistic behavior,” was employed by Oakes and colleagues to examine the profile of RRBs in 39 boys with FXS, aged 6–10 years, without specifying their ASD status [194]. Restricted Interests and Sensory Motor behaviors were reported as most problematic, in contrast to SIB. Non-verbal IQ was negatively related to RRBs in general, whereas anxiety and social affective symptoms of ASD were positively correlated with scores on Restricted Interests. Anxiety was also positively correlated with scores of Compulsive behaviors and Ritualistic Sameness behaviors. Despite the relative specificity of the reported profile [194], it is important to point out that all subscales of the RBS-R were significantly inter-correlated. No application of the RBS-R for measuring changes in non-ASD-related RRBs has been reported. Therefore, this particular use of the RBS-R cannot be evaluated or a tool quality grading of *no evidence available* is the most appropriate.
2. ABC-C Stereotypy and Inappropriate Speech subscales. These are two of the five original subscales of the ABC-C that cover RRBs. The content of both subscales was relatively preserved after the factor analysis that originated the ABC-C_FX_ (i.e., one Stereotypy item was incorporated into the new Socially Unresponsive/Lethargic subscale; [111]), demonstrating their appropriateness for FXS. Two independent studies demonstrated the usefulness of these subscales for delineating trajectories of RRBs in FXS [103, 111]. While these cross-sectional analyses showed that behaviors under Stereotypy are in the moderate range of severity and tend to slightly decrease over time, those under Inappropriate Speech are on the severe range and remain relatively stable between 6 and 25 years. Since the ABC-C_FX_ autistic behavior-oriented Socially Unresponsive/Lethargic subscale did not incorporate many items of Stereotypy and Inappropriate Speech, it is assumed that these subscales reflect predominantly non-autistic RRBs. However, no direct evidence supporting this is currently available. As the ABC-C_FX_ Inappropriate Speech subscale includes only four items; this may limit its sensitivity in a similar fashion to the Social Avoidance subscale (see the “Anxiety” subsection, under the “Behavior and Emotion measures” section). We can conclude that, as other ABC-C_FX_ subscales, Stereotypy and Inappropriate Speech are adequate for assessing either *shorter*-*term* or *longer*-*term* outcomes. Psychometric data, including their application to several recent FXS trials, support a *moderate* general tool quality label. Nonetheless, no data are available on the specific use of these instruments for measuring non-autistic RRBs in FXS. Therefore, their tool quality for this specific indication would correspond to *no evidence available*.

#### Potential measures

1. Repetitive Behavior Questionnaire (RBQ). The RBQ is a 19-item scale that shares some individual items with the RBS-R. Moss and colleagues examined the presence of RRBs across six groups of individuals with ID, including males with FXS (*n* = 191), aged 6–47 years. It was reported that the FXS group had higher scores on all five subscales (Stereotyped Behavior, Compulsive Behavior, Insistence on Sameness, Restricted Preferences, and Repetitive Speech); moreover, they also had more severe RRBs than two other ID groups in the categories of Compulsive Behavior, Insistence on Sameness, and Repetitive Speech [189].
2. Behavior Problems Inventory (BPI). Employing an earlier version of the BPI scale [195], an instrument described in the “Problem behaviors: focus on disruptive behavior domain” subsection, and the RBS, Bodfish and colleagues found that the difference in RRBs between ID and ASD was mainly quantitative [179]. Namely, they reported that the occurrence of specific topographies of RRBs as well as their severity in 34 adults (23 males, 11 females) with non-ASD ID (CARS <30) matched to that of an ASD group. This finding supports the notion that the RRBs in ASD are similar to those described in ID [196]. While both groups, ID and ASD, had significant patterns of RRBs co-occurrence, the exception was the frequency of dyskinesias that was higher in the ASD group.

Although the data on the RBQ and the earlier version of the BPI support the notion that instruments measuring RRBs in ASD are adequate for FXS and other forms of ID, no data on the use of these instruments in clinical trials in FXS is available.

#### Conclusions

While the RBS-R and the ABC-Cfx Stereotypy and Inappropriate Speech subscales present many adequate attributes and they have been already applied to intervention studies in FXS, their adequacy for measuring RRBs other than autistic behaviors has not been formally assessed. Therefore, further testing other suitable measures, such as the RBQ and BPI, or developing new ones could still be a worthwhile effort.

#### Overall conclusions on Behavior and Emotion measures

While the main targets of intervention studies in FXS have been maladaptive/problem behaviors, and these overlap with ADHD-like behaviors and RRBs, anxiety and autistic behaviors are also a major concern. Over the years, this situation has raised the question whether it is possible to develop a comprehensive instrument covering all abnormal behaviors in FXS. The ongoing application and refinement of the FXSRS [114], during the course of trofinetide trials in FXS, represents the most recent effort in this area. This instrument is a traditional clinician rating scale. While it is too early to determine the value of the FXSRS, the utility of a single “everything” measure is obvious. Nonetheless, the pervasive use of devices such as smart phones, tablets, and wearable sensors in behavioral and mental health research [197, 198] begs the question of when would be appropriate to incorporate technologies to behavioral assessments in FXS, including in the context of clinical trials. These novel technologies may allow more naturalistic, situation-related, and multiple sampling of behavioral abnormalities, perhaps with greater implications for quality of life, in FXS and other neurodevelopmental disorders. Thus, paradigms for adequate validation of such novel measures would be an important contribution to the field.

Finally, the issue of biologic validation of behavioral measures, recommended in the 2013 Report through correlations with valid FXS biomarkers, remains pending due to the slow progress in the Biomarkers/Medical domain as reviewed in the following domain and in the “Discussion” section.

## Biomarkers and Medical measures

The Working Group endorsed further effort toward validating existing biomarkers as critically needed measures for development in the field. Their objective nature and closer link to CNS function make them particularly attractive as outcome measures. The Biomarkers group includes a wide range of measures, from the relatively easy-to-apply blood-based biomarkers to those providing more direct evidence of CNS function such as neurophysiological and neuroimaging methods. In spite of their wide application, none had well-established clinical correlations or validity at the time of the 2013 Report.

Table 5 lists potential biomarkers classified in terms of their suitability for *shorter*- vs. *longer*-*term* outcomes and COSMIN quality grading system.

**Table 5:** Potential biomarker measures in fragile X syndrome

Domains	Potential outcome Measure	*Shorter term*	*Longer term*	Quality of tools
Blood and tissue				
*FMR1*/FMRP expression^a^	*FMR1* methylation	Yes	Yes	+ [9, 113, 261]
	FMRP levels	Yes	Yes	+ [9, 113]
Signaling pathway				
	ERK activation rate	Yes	Yes	+ [54, 55, 200, 203]
	BDNF level	Yes	Yes	+ [159, 204, 208]
	APP and metabolites levels	Yes	Yes	+ [159, 209]
	MMP activity	Yes	Yes	+ [87, 227]
	mTOR activity	Yes	Yes	+ [269]
	S6 Kinase activity	Yes	Yes	+ [269]
Neurophysiological				
	Electroencephalogram	Yes		+[217, 218, 227, 228, 232, 233]
	Event-related potentials	Yes	Yes	+/++ [219, 222–224, 227–230]
	Prepulse inhibition (PPI)	Yes		+/++ [6, 117, 226]
	Eye tracking and pupillometry	Yes		++ [77, 85–88, 139, 216]
Neuroimaging	MRI			
	sMRI	Yes	Yes	+ [235]
	-vMRI	Yes	Yes	+ [237–240]
	-DWI	Yes	Yes	+ [241–243]
	Functional MRI			
	-fMRI	Yes	Yes	+ [244–250]
	-rs-fMRI	Yes	Yes	+ [77]
	-MRS	Yes	Yes	+ [270]
	-pMRI	Yes	Yes	+ [234]
	NIRS	Yes		+ [251–255]

*Abbreviations*: *FMR1* fragile X mental retardation 1 gene, *FMRP* fragile X mental retardation protein, *ERK* extracellular signal-related kinases, *BDNF* brain-derived neurotrophic factor, *APP* amyloid precursor protein, *MMP* matrix metalloproteinases, *mTOR* mechanistic target of rapamycin, *MRI* magnetic resonance imaging, *pMRI* perfusion MRI, *sMRI* structural MRI, *vMRI* volumetric MRI, *DWI* diffusion-weighted imaging, *fMRI* functional MRI, *rs-fMRI* resting state fMRI, *MRS* magnetic resonance spectroscopy, *NIRS* near-infrared spectroscopy, *Shorter term* deemed suitable for clinical trials lasting <12 months, *Longer term* deemed suitable for clinical trials lasting >=12 months. Grading system: *+++* strong evidence, *++* moderate evidence, *+* limited evidence, *?* unknown due to poor methodological quality, *blank cell* no evidence available

^a^Evaluated as a predictor of response

### Blood and tissue biomarkers

#### Background

Deficient FMRP expression has significant downstream consequences disrupting many cellular signaling pathways. As mentioned above, this is a particularly attractive group of measures because of their minimally invasive nature and easy application.

#### Progress and plans in FXS

Blood-based analyses hold promise as minimally invasive windows into cellular dysregulation secondary to deficient FMRP production occurring both in the periphery and, at least some, regions of the brain. Several molecular and biochemical blood assays have been evaluated in the context of published FXS-specific drug trials.

1. Mitogen-activated protein kinases/extracellular signal-regulated kinases (MAPK/ERKs). The MAPK/ERKs are a group of proteins that regulate nodal points for several signaling cascades. MAPK/ERK regulation mediated by phosphorylation is typically termed activation. Delayed early-phase phosphorylation of ERK has been noted in lymphocytes of individuals with FXS and in neurons and thymocytes of *Fmr1* knockout mice. This delay is described as an enhanced time to half maximum ERK activation following phorbol ester stimulation [199]. ERK peripheral lymphocytic activation has been assessed pre- and post-drug treatment in studies of lithium and riluzole in FXS. ERK activation was significantly reduced (i.e., normalized) following lithium [55] or riluzole [200] administration. In an open-label trial involving 16 individuals with FXS, aged 6–23 years, lithium (a mood stabilizer that inhibits the phospholipase C signaling pathway and GSK-3B [201]) use was associated with normalization of ERK activation kinetics but no improvement in maladaptive behaviors evaluated by the ABC-C [54]. These findings can be explained, at least in part, because of the high variability of the biochemical assay (Berry-Kravis, personal communication). Riluzole is FDA approved for treatment of amyotrophic lateral sclerosis and is postulated to reduce glutamate release, block NMDA receptors, and enhance GABA activation [202]. In this pilot 6-week trial including six males with FXS, aged 19–24 years, Erickson and colleagues found that despite normalization of ERK activation kinetics, no significant clinical improvements were noted [200]. A recent evaluation of ERK and Akt (another major kinase pathway) phosphorylation in individuals with FXS, in relation to lovastatin treatment, demonstrated normalization of baseline increased ERK activity after drug administration. Of note, these changes in ERK phosphorylation were correlated with clinical response to lovastatin [203].
2. Brain-derived neurotrophic factor (BDNF). BDNF is a growth factor and synaptic modulator that supports survival, growth, and differentiation of neurons. BDNF has been shown to impact FMRP expression [204]; its application to hippocampal slices from *Fmr1* knockout mice leads to rescue of long-term potentiation deficits [205]. BDNF expression has also been noted to be reduced in *Fmr1* knockout mice [206]. In a study of acamprosate in 12 individuals with FXS, aged 5–17 years, BDNF plasma levels were shown to increase significantly after 10 weeks of treatment with the drug [159]. Nonetheless, BDNF levels in the nine subjects deemed to be responders did not correlate with their clinical response. Acamprosate is drug approved for the treatment of alcoholism, which is postulated to attenuate glutamatergic activity, particularly NMDA receptor-dependent, and potentiate GABA A receptor activity [207]. Additional relevant data on BDNF were obtained in a small open-label fluoxetine trial in children and adolescents with idiopathic ASD. Using the Autism Treatment Evaluation Checklist, the cohort showed improvements in several aspects of communication, socialization, and cognitive awareness, changes that were correlated to decreases in serum levels of BDNF [208]. Since the direction of the BDNF changes was unexpected, this study emphasizes the need for further exploration of BDNF and related molecules as biomarkers of response to treatment in neurodevelopmental disorders including FXS.
3. Amyloid precursor protein (APP). APP is a key neural modulator, which is processed by two pathways. The best known is the so-called amyloidogenic or β pathway that leads to the production of the neurotoxic Aβ40 and Aβ42 fragments, which have been implicated in the pathophysiology of Alzheimer disease. The non-amyloidogenic or α pathway results in the production of the soluble neurotrophic APP alpha (sAPPα) [209]. FMRP is known to regulate APP mRNA expression [210, 211], with baseline APP levels being elevated in *Fmr1* knockout mice [211]. APP and its cleavage products were measured in a subset of nine participants (mean age 10.9 years) in the acamprosate trial described above [159]. Total APP and sAPPα were reduced after treatment, while Aβ40 and Aβ42 remained unchanged. These APP-related decreases correlated modestly with improvements on the ABC-C Lethargy/Social Withdrawal subscale.

#### Potential measures

A variety of blood-based biomarkers are being considered in translational research in the idiopathic ASD field. They include among others markers of immune processes [212], inflammation [213], oxidative metabolism [214], and the serotoninergic system [215]. It is possible that, as BDNF and APP, these ASD-related biomarkers will become eventually applicable to FXS trials.

#### Conclusions

The body of literature on blood biomarkers in FXS continues to increase; however, changes are still inconsistent or unrelated to clinical effects. Only the ERK activation assay has been replicated, and most studies have involved small cohorts. Not only larger-scale replication investigations but also studies exploring other biomarkers are needed, since the impact of deficient FMRP are wide-reaching and likely affecting many cellular pathways that could be potentially evaluated in peripheral blood. Thus, in terms of tool quality, we consider blood-based biomarkers *limited* (BDNF, amyloid markers, ERK). Because of their dynamics, these biomarkers are adequate for evaluating *shorter*-*term* effects in FXS. Nevertheless, follow-up studies may determine their appropriateness as *longer*-*term* measures.

### Neurophysiological measures

#### Background

There is a critical need for objective direct measures of CNS function that can provide feedback about a drug’s engagement to its target. Although potentially valuable as non-invasive measures of treatment response, blood biomarkers may not directly reflect CNS function, as receptors and other molecules targeted by drugs are frequently either not expressed in blood cells or linked to different signaling cascades than in the CNS. The Working Group recommended (1) to correlate biomarkers that measure brain activity directly such as prepulse inhibition of the startle response (PPI), eye tracking/pupillometry, and functional MRI (fMRI) with measures of neurobehavioral function in individuals with FXS in order to establish clinical validity [6]. The need for new markers of CNS engagement has been made even more apparent by the recent challenges in seeing significant changes in behavior with drugs targeting synaptic dysfunction in FXS (“failed trials”).

In the 2013 Report, it was noted that PPI was the most extensively studied electrophysiological measure of relevance to clinical trials in FXS. PPI reflects sensory inhibitory gating, which is regulated by mGlur5 signaling and deficient in FXS [117]. In addition, PPI has good test-retest reliability and a similar profile in individuals with FXS and *Fmr1* knockout mice [117]. All these features make PPI a promising biomarker in FXS.

Eye gaze abnormalities are well documented in FXS; gaze avoidance is a hallmark behavioral feature of the disorder, with affected individuals having difficulty in establishing and maintaining eye gaze during social interactions [2]. Pupillary responses, regulated by the autonomic nervous system in part in response to level of emotional arousal, are also abnormal in FXS [85, 216]. Eye-tracking devices such as the Tobii system have been successfully used to measure eye gaze aversion and pupillary responses in FXS [85, 216], supporting the notion that these could become biomarker-type of outcome measures in clinical trials.

Electroencephalogram (EEG) is one of the oldest non-invasive methods for evaluating brain function, by measuring on the scalp fluctuations in electrical activity that reflect neural network properties [217]. Despite its poor spatial resolution, EEG continues to be a valuable tool for research where millisecond-range temporal resolution is desired [218]. In the clinical context, one type of EEG parameter typically estimated with potential translational research use is spectral content (e.g., spectral power of different frequency bands) [210]. Other commonly used derivatives of EEG signal used in clinical and cognitive research, with translational potential, include evoked potentials (EPs) and event-related potentials (ERPs). EPs are estimated by averaging over EEG activity that is time-locked to the presentation of a stimulus (e.g., auditory tone or visual checkerboard). Similarly, ERPs are estimated by averaging over EEG activity that is time-locked to more complex higher-order cognitive processing of the stimuli (e.g., ERP related to response inhibition in a Go/No-Go experimental paradigm). In FXS, auditory ERP is known to be abnormal with a larger magnitude of response [219] in correspondence with the characteristic behavioral phenomenon of sensory over-reactivity (hyperarousal) to sound [220]. Over-reactivity to a variety of sensory stimuli is common in FXS and thought to represent circuit hyperactivation associated with increased dendritic spine density in sensory cortices [2]. A barrel cortex neuronal model for this phenotype has been described in the *Fmr1* knockout mouse [221]. As individuals with FXS, *Fmr1* knockout mice also show a larger auditory response amplitude [222]. Thus, auditory ERP may not only be a direct window into synaptic dysregulation in FXS but also a highly attractive measure for direct translation of drug effects from mouse to man. ERP measures may also be relevant to the distinctive mGluR5 dysregulation in FXS, since the mGluR5 antagonist MPEP reduced N1 auditory EP amplitudes in a mouse model of ASD [223]. There is also evidence that ERP resting-state measures are modulated by inhibition of mGluR5 signaling by the mGlur5 antagonist mavoglurant [224]. Another ERP parameter, mismatch negativity (MMN) may also be a useful measure in FXS trials, as it has been reported to be an index of language impairment in ASD [225]. Additional information about the application of EGG-related to measures to FXS and background on PPI and eye tracking/pupillometry is included in the following section.

#### Progress and plans in FXS

1. Prepulse inhibition (PPI). The potential of PPI as a biomarker in FXS was confirmed in an early trial (*n* = 12) with the mGluR5 antagonist fenobam that showed a ≥ 20% improvement in half of the subjects (although there was no clear clinical improvement) [226]. However, subsequent work with PPI reveals that the measure is very sensitive to differences in equipment and environment, which has led to lack of inter-site reproducibility. Consequently, PPI may be most useful in early proof-of-concept trials as opposed to large confirmatory multi-site trials. PPI seems suited for evaluating *shorter*-*term* effects, and its tool quality is *limited*-*moderate* for the aforementioned reasons.
2. Eye tracking and pupillometry. A standardized protocol quantifying pupillary diameter and looking time and number of fixations to the eye region for a series of happy, calm, and fearful human faces has been shown to be suitable for individuals of varying ages with FXS, with a 7–51 years age range, with strong feasibility and test-retest reliability for all outputs [85]. This protocol has been applied in several clinical trials, although a full analysis of sensitivity to treatment has not yet been published. However, preliminary reports indicate normalization of parameters in response to treatment with minocycline [87] or the mGluR5 antagonist mavoglurant [88]. The eye-tracking protocol described above is the only one employed in clinical trials in FXS; nonetheless, several others have been developed and are potentially useful (see the “Social cognition” section). In conclusion, although reports on ongoing studies may change our conclusions, at present, eye tracking and pupillometry should be considered *moderate* level quality tools suited for evaluating *shorter*-*term* effects in FXS trials.
3. Event-related potentials (ERPs). Several observational and treatment studies, mainly pilot type, have utilized EEG to characterize ERPs in FXS and to assess treatment response and associated changes in cortical activity. Van der Molen et al. showed that the N1 and P2 components of auditory ERPs from 16 males with FXS, aged 18–42 years, were significantly enhanced. Schneider and colleagues observed changes in electrocortical habituation to auditory stimuli (changes between the first and last of 45 presented stimuli), specifically improved N1 and P2, associated with minocycline treatment (double-blind, placebo-controlled crossover, *n* = 12, mean age 10.5 years), suggesting the feasibility and sensitivity of ERPs as a biomarker in FXS treatment trials [227]. In another study, Yang et al. employed auditory ERPs to evaluate the effects of chronic memantine treatment on verbal memory in individuals with fragile X-associated tremor/ataxia syndrome (FXTAS) [228, 229]. The authors reported treatment-associated benefit on cued-recall memory as well as corresponding changes in N400 ERP response [228, 229]. Recently, Ethridge and colleagues also examined ERPs during a somewhat different passive auditory habituation task in individuals with FXS (*n* = 14, 14–57 years) and found “giant” N1 amplitudes to all four repeated stimuli in the FXS group more frequently than in a matched control subjects [230]. Of relevance to behavioral measures in FXS, the N1 amplitude enhancement was strongly correlated with auditory processing abnormalities on the Sensory Profile, a caregiver questionnaire employed in the ASD field that measures children’s sensory processing abilities [231], and with increased ASD features on the SCQ [230]. N2 ERP amplitudes were also decreased in FXS and correlated with scores on the ABC-C Irritability subscale. Overall, this study [230] exemplifies the type of information provided by auditory ERPs in FXS that links biomarkers and behavioral outcome measures. No study so far has examined age- or gender-related differences in ERP profiles in FXS, and no data are available on test-retest reliability or other measurement properties. However, studies are currently in progress to examine resting state and MMN in FXS. The use of ERP as a biomarker of multiple aspects of neural activity and processing in FXS is currently being explored at several centers. This includes the introduction of auditory ERP as a biomarker in the upcoming mavoglurant and language learning trial in children with FXS (U01 NS096767). Thus, at present, auditory ERP seems to be a biomarker suited to evaluating medication *shorter*- or *longer*-*term* effects, with current evidence supporting a *limited*-*moderate* tool quality. Other potentially valuable neurophysiological measures, in particular EEG spectral analyses, are just beginning to be examined in FXS, as described in the following section.

#### Potential measures

##### EEG spectral analyses.

These are being developed, and the recent pilot application of the frontal alpha band asymmetry index, a measure linked to anxiety and mood disorders, in a Rett syndrome trial testing full-length IGF-1 (mecasermin) suggests a good potential for EEG spectral analyses in FXS. IGF-1 treatment not only markedly reduced the right-sided abnormal asymmetry present in most subjects but these changes also correlated with reduction in anxiety severity [139]. Pilot studies have reported that gamma band amplitude and asymmetry could be biomarkers of response to behavioral interventions in ASD [232, 233], and studies to evaluate this parameter are ongoing in FXS.

#### Conclusions

PPI is a biomarker with utility limited to early-phase trials. Both eye tracking and pupillometry are biomarkers of greater potential because they may be applicable throughout the continuum of clinical drug development. EEG-related measures such as auditory ERP and spectral analyses have great potential in FXS and are beginning to be tested in FXS trials. Other physiological measures already explored in neurodevelopmental disorders, such as actigraphy (accelerometer) studies of hand stereotypies in ASD and Rett syndrome, will be hopefully extended to FXS.

### Neuroimaging studies

#### Background

Magnetic resonance imaging (MRI) is a neuroimaging modality that uses magnetic field and radiofrequency waves for in vivo imaging of the CNS. Structural MRI techniques include volumetric MRI (vMRI) and diffusion-weighted imaging (DWI) for mapping the morphology and white matter microstructure of the brain, respectively. Functional techniques include fMRI for measuring blood oxygenation level dependent (BOLD) signal, magnetic resonance spectroscopy (MRS) for measuring metabolic changes in the brain, and perfusion MRI (pMRI) for examining tissue perfusion [234]. A large number of MRI studies of individuals with FXS demonstrate that brain structure, function, connectivity, and metabolism are abnormal beginning early in development. For a review, see Fung and Reiss [235]. Some of these studies were conducted on a longitudinal basis, thereby providing initial “growth curves” of brain development in FXS, already available for typical development and other disorders [236]. Comparisons of brain growth curves from individuals with FXS with normative data could assist in the characterization of cohorts, including profiling responders, as well as providing information about treatment response in future trials.

#### Progress and plans in FXS

1. Structural MRI. Research in other disorders indicates that significant changes in gray and white matter volume, white matter microstructure, and brain activation can occur in association with intervention within relatively short periods of time. For example, Roberto and colleagues showed that short-term (mean 50 days) weight restoration in under-weighed adult patients with anorexia nervosa was associated with significant increases in gray and white matter volume relative to healthy controls [237]. Eijk et al. demonstrated that, in individuals with alcohol abuse, 2 weeks of supervised abstinence was associated with significant recovery of gray matter volume in several brain regions including the cerebellum and parietal lobe [238]. There are many other examples of relatively rapid changes in neuroimaging metrics in clinical groups receiving disorder-focused intervention. Administration of risperidone and ziprasidone to individuals with schizophrenia led to increased cerebral gray matter volume after 28 days of treatment [239] and administration of citalopram to depressed patients increased hippocampal gray matter within 8 weeks [240]. Changes have also been observed in white matter microstructure by DWI; patients with multiple sclerosis showed increases in fractional anisotropy within 2 months of facilitation of physiotherapy [241], depressed patients showed increase in fractional anisotropy with 4 weeks of psychotherapy [242], and patients with obsessive-compulsive disorder showed a reversal of abnormal white matter microstructure after a 12-week course of antidepressant (SSRI) treatment [243].
2. Functional MRI. The feasibility and utility of fMRI as a putative biomarker of intervention has been demonstrated in hundreds of treatment studies, as described in recent reviews of pharmacology, psychotherapy, pain management, and rehabilitation [244–250]. A recently completed study by Reiss and colleagues utilized fMRI as an outcome measure in a clinical trial of donepezil for FXS; these functional imaging data are currently being analyzed to determine if drug-related effects are observable and associated with clinical change.

#### Potential measures

Near-infrared spectroscopy (NIRS) is a method of diffuse optical brain imaging that uses light to non-invasively probe the cerebral cortex for changes in blood oxygenation related to brain function [251]. NIRS-based neuroimaging provides excellent temporal sensitivity, as well as reasonable spatial sensitivity. NIRS is relatively easy to set up, portable, and more tolerant to movement than MRI. Technological improvements and advancement in signal processing methods now enable scientists to employ NIRS to investigate a variety of hard-to-test clinical populations within naturalistic environments. The aforementioned advantages have resulted in the use of NIRS as a tool to monitor brain function related to clinical conditions including epilepsy, migraine, cerebrovascular disease, schizophrenia, ADHD, and others [252]. There is also emergent use of NIRS as an outcome measure in intervention studies in neurorehabilitation [253]. For example, NIRS-based brain-computer interface (BCI) systems, which establish a direct connection between the brain and an external device, have been successfully used in clinical trials of movement disorders and paralysis [254]. Neurofeedback applications, which provide clinical populations with (near) real-time information about their own brain functioning, have demonstrated long-term improvement in focused brain activity [255]. For patients with acquired or congenital brain insult, where elicitation of focused brain activity may be a crucial step toward complete neurorehabilitation, NIRS-based neurofeedback affords a potentially valuable treatment tool as well as an outcome measure. Thus, although not directly in FXS, data on NIRS supports its potential as a biomarker for evaluating *shorter*-*term* effects of interventions.

#### Conclusions

In conclusion, MRI techniques are potential biomarkers adequate for evaluating *shorter*- and *longer*-*term* effects. Due to their *limited* application in clinical trials in neurodevelopmental disorders, it is not possible at this point to assess the tool quality of MRI and other neuroimaging techniques. Nevertheless, due to its high cost and complexity, neuroimaging would be probably best employed in early-phase and target engagement studies in FXS.

#### Overall conclusions on Biomarkers and Medical measures

These are promising tools; however, the body of evidence supporting their use as outcome measures is still small. Despite this, the unique properties of biomarkers (e.g., objectivity, direct demonstration of target engagement) suggest that their inclusion in future studies developing endpoints or in early-phase intervention studies is critical.

## Other outcome measures

Additional categories of outcome measures are being considered in FXS. The most advanced at present are instruments for assessing motor function, specifically the neuromotor battery under development by Tartaglia and colleagues [256].

In contrast to other neurodevelopmental disorders (e.g., Rett syndrome), impairments in FXS have been typically measured in a domain/area-specific function (e.g., language, anxiety). To date, no scales of overall clinical severity have been applied systematically to FXS. Nonetheless, a re-evaluation of the role of disorder-specific features and behavioral phenotype [3] is taking place, particularly in the context of clinical trials. For instance, the co-occurrence of different behavioral conditions (e.g., ASD and anxiety) in individuals with FXS modifies their presentation, evaluation, and interpretation [28]. Thus, the FXSRS [114], which covers a wide range of specific and common behavioral symptoms in FXS (discussed in the “Problem behaviors: focus on disruptive behavior domain” section), is the first attempt at developing a clinical severity scale for measuring response to interventions in FXS. Whether non-behavioral features should be added to measures such as the FXSRS will depend on the evaluation of their functional impact [43], a process at an early stage in FXS [17]. Indeed, as reviewed in the “Discussion” section, development and evaluation of quality of life measures in observational and intervention studies has just begun in FXS [257, 258].

## Discussion and conclusions

FXS is a complex multi-system neurodevelopmental disorder. Its neurobehavioral manifestations, which include variable cognitive and language impairments, are linked to functional impairments and reduced quality of life [2]. FXS became a prototype neurodevelopmental disorder for developing neurobiologically targeted treatments (i.e., treatments targeting neurobiological abnormalities resulting from the primary genetic defect). Its well-characterized genetics, advanced neurobiological knowledge, and availability of animal models, in conjunction with increasing work on psychopharmacology, made FXS a focus of attention for federal funding and regulatory agencies and other stakeholders interested in developing new treatments. Two meetings led to the formation of Working Groups of experts and a published report on outcome measures in FXS [6]. Despite the relatively short interval between the 2013 Report (an updated version of the conclusions of the 2009 meeting) and the present review, the intense activity particularly in the field of targeted clinical trials and the apparent failure of all drug development programs that have advanced to phase IIb/III have made necessary this update on outcome measures in FXS.

The 2013 Report’s overall conclusions were that in all three domains/areas, Cognition, Behavior/Emotion, and Biomarkers/Medical, there was a need for additional validation of existing and development of new measures for FXS. Of the available outcome measures, those covering the behavioral domain and in particular rating scales and questionnaires were closer to meeting all criteria for optimal endpoint. Specific recommendations were made for each area and type of measure. The present revision takes advantage of the additional experience collected in the last few years, a period in which further application of “older” measures and development and validation of newer instruments took place mainly in the context of ongoing intervention studies. Our overall conclusion is that the general and most of the specific recommendations of the 2013 Report are still valid [6]. Nonetheless, two areas have seen some steady progress: Cognition and Behavior/Emotion. In these fields, measures have been either adapted or developed specifically for FXS. With more ongoing and planned work on cognitive and behavioral instruments, the future seems encouraging. On the other hand, the Biomarkers/Medical area with its promise of providing more objective and quantitative tools has developed slowly, to some extent because of the reluctance of industry to embark on complicated and expensive projects that may not meet regulatory approval. The FXS clinical community can help with this process, by implementing in the clinic novel outcome measures, particularly those in the category of biomarkers. It is the continuous use and validation in the clinical setting that will make new tools more acceptable as meaningful endpoints. Thanks to the FXCRC, the FXS community is in a unique position for carrying out this endeavor.

Although the general *conclusions* of the present report suggest that optimal outcome measures for FXS are not yet fully developed, these conclusions should be seen as motivation for additional work [259, 260]. Recent similar efforts by the UK’s NHS National Institute for Health Research [11] and Autism Speaks [13–15], which have evaluated mainly behavioral outcome measures for idiopathic ASD, have arrived at the same conclusions: few instruments meet validation criteria. The main question is whether this situation should lead to halting clinical trials while the shortcoming is remediated. While this may be an option for cognitive and behavioral trials, development of biomarkers is by nature slow, expensive, and more appropriate as a component of a clinical trial. Moreover, evaluating sensitivity of outcome measures is virtually impossible without their incorporation into intervention studies. Some information can be inferred from natural history studies and other investigations measuring change over time. However, the definitive answer can only be obtained from pragmatic (clinic practice-based) or conventional research clinical trials. The complications of sensitivity assessments are underscored by their dependence on treatment effectiveness (e.g., borderline effect of a drug at a given dose in FXS, but clear effect in another neurodevelopmental disorder). All of this means that refinement of existing and development of new outcome measures and biomarkers will be a continuing process incorporated to ongoing and future FXS trials and associated with surveillance of related work in other neurodevelopmental disorders [229]. This seems the only feasible approach; however, clinicians, scientists, participants, their families, and advocacy groups involved in FXS clinical trials should understand that, with this strategy, it is likely to take a number of trials to get study design worked out before a successful registration effort is achieved. Working closely with an informed FXS community seems the best strategy, if we continue moving forward with targeted trials while developing concurrently outcome measures.

An additional issue raised by the 2013 Report and the British and American efforts in ASD is the need to follow the recommendations of these reviews. Paraphrasing the 2013 Report, we should aim at “using a more consistent battery of measures across trials” in FXS. Clinical networks provide the ideal framework for the implementation of such batteries of outcome measures. The creation of a Clinical Trials Committee in the FXCRC could be seen as a step toward this goal. The committee is the most adequate entity for integrating clinical networks, such as the NIH-funded NeuroNEXT, drug companies, and other stakeholders involved in the development of clinical trials. FDA validation of an outcome measure for a given clinical trial has the benefit that such endpoint could be used in future studies, even if they do not involve the same drug. Thus, coordinated work among FXS clinical trial investigators may also lead to the availability of validated outcome measures for multiple clinical trials and for comparing the potency of different pharmacological and non-pharmacological interventions.

Finally, it is important to remember that in recent years, the FDA and its European counterpart, the European Medicines Agency, have emphasized the need not only to go beyond the measurement properties of endpoints but to also consider their meaning in terms of quality of life. In FXS, the common use of adaptive behavior instruments (e.g., Vineland-II) has helped to provide information on the potential functional implications of a “positive” trial [6]. However, the systematic use of adaptive behavior and other more direct measures of quality of life in the validation of outcome measures in FXS would be an essential step as it has already been for other neurodevelopmental disorders [17]. Initial work with the ABC-C in FXS demonstrates the value of such endeavor [257, 258]. Ultimately, more solid outcome measures will benefit not only the development of new pharmacological interventions but of all types of treatments in FXS.

## Abbreviations

ABC-C: Aberrant Behavior Checklist-Community Edition
ABC-C_I: ABC-C Irritability subscale
ABC-C_SW: ABC-C Lethargy/Social Withdrawal subscale
ABC-C_T: ABC-C total score
ABC-C_FX_: Aberrant Behavior Checklist-Community Edition FXS-specific
ABC-C_FX__SA: ABC-C_FX_ Social Avoidance subscale
ACTeRS: Attention-Deficit Hyperactivity Comprehensive Teacher’s Rating Scales
ADAMS: Anxiety Depression and Mood Scale
ADAMS_T: ADAMS total score
ADHD: Attention-deficit hyperactivity disorder
ADHD-RS: Attention-Deficit Hyperactivity Disorder Rating Scale
ADHD-RS-IV Ed: ADHD Rating Scale-IV
ADI-R: Autism Diagnostic Interview-Revised
ADIS: Anxiety Diagnostic Interview Scale for DSM-IV
ADOS: Autism Diagnostic Observation Schedule
ADOS-CSS: ADOS-Calibrated Severity Score
ADOS-G: ADOS-Generic
AFQ056: Mavoglurant mGluR5 receptor antagonist
ALR: Allan L. Reiss, M.D.
APP: Amyloid precursor protein
ASD: Autism spectrum disorder
ATN: Autism Treatment Network
BASC: Behavioral Assessment for Children Scale
BCI: Brain-computer interface
BDNF: Brain-derived neurotrophic factor
BOLD: Blood oxygenation level dependent
BPI-S: Behavior Problems Inventory Short Form
CAG: Craig A. Erickson, M.D.
CARS: Childhood Autism Rating Scale
CASI-4R: Child and Adolescent Symptom Inventory-4th Ed Revised
CBCL: Child Behavior Checklist
CBI-M: Caregiver Burden Inventory-Modified
CELF-5/P2: Clinical Evaluation of Language Fundamentals-5th Ed/Preschool-2nd Ed
CGI-I: Clinical Global Impression-Improvement
CGI-S: Clinical Global Impression-Severity
CNS: Central nervous system
CNT: Contingency Naming Task
Conners Abb: Conners Abbreviated Version
Conners GI-P: Conners General Index-Parent Version
COSMIN: COnsensus-based Standards for the selection of health Measurement INstruments
CSHQ: Children’s Sleep Habits Questionnaire
CSQ: Caregiver Strain Questionnaire
DAMP: Dextroamphetamine
DBB: Dejan B. Budimirovic, M.D.
DBC: Developmental Behavior Checklist
DH: David Hessl, Ph.D.
DQ: Development quotient
DSM-5: Diagnostic and Statistical Manual of Mental Disorders-5th Edition
DSM-IV: Diagnostic and Statistical Manual of Mental Disorders-IVth Edition
DWI: Diffusion-weighted imaging
EBK: Elizabeth Berry-Kravis, M.D. Ph.D.
EEG: Electroencephalogram
EF: Executive function
ELS: Expressive language sampling
ERK: Extracellular signal-related kinases
ERP: Evoked response potentials
ET: Eye tracking
EVT-2: Expressive Vocabulary Test 2nd ed
FDA: Food and Drug Administration
FMP: Fast-mapping performance
*FMR1*: *Fragile X mental retardation 1* gene
fMRI: Functional MRI
FMRP: Fragile X mental retardation protein
FSIQ: Full-scale intelligence quotient
FXCRC: Fragile X Clinical and Research Consortium
FXS: Fragile X syndrome
FXSRS: Fragile X Syndrome Rating Scale
FXTAS: Fragile X-associated tremor/ataxia syndrome
GABA: *g*-aminobutyric acid
HR: Heart rate
HRV: Heart rate variability
HSQ-PDD: Home Situations Questionnaire—PDDs version
ID: Intellectual disability
IGF-1: Insulin-like growth factor
ILS: Independent Living Scales
IQ: Intelligence quotient
KiTAP: Tests of Attentional Performance for Children
LA: Leonard Abbeduto, Ph.D.
MAPK/ERK: Mitogen-activated protein kinases/extracellular signal-regulated kinases
MASC: Multidimensional Anxiety Scale for Children
MDX: Metadoxine
mGluR5: Metabotropic glutamate receptor 5
mGluRs: Group I metabotropic glutamate receptors
MKK: Margaret K. King
MMN: Mismatch negativity
MMP9: Matrix metallopeptidase 9
MPH: Methylphenidate
MRI: Magnetic resonance imaging
MRS: Magnetic resonance spectroscopy
MSEL: Mullen Scales of Early Learning
mTOR: mechanistic target of rapamycin
NICHD: National Institute of Child Health and Human Development
NIH: National Institutes of Health
NIH-TCB: NIH Toolbox Cognitive Battery
NIRS: Near-infrared spectroscopy
PARS: Pediatric Anxiety Rating Scale
PDD: Pervasive Developmental Disorders
PI: Principal Investigator
PK: Pharmacokinetics
PLS-5: Preschool Language Scale-Version 5
pMRI: Perfusion MRI
PPI: Prepulse inhibition
PPVT-4: Peabody Picture Vocabulary Test-4th Ed
PSI: Parenting Stress Index
RBANS: Repeatable Battery for the Assessment of Neurological Status
RBQ: Repetitive Behavior Questionnaire
RBS-R: Repetitive Behavior Scale-Revised
RCADS: Revised Children’s Anxiety and Depression Scale
RSA: Respiratory sinus arrhythmia
rs-fMRI: Resting state fMRI
sAPPα: Soluble neurotrophic amyloid precursor protein alpha
SB5: Stanford-Binet Version 5
SBS: Stereotyped Behavior Scale
SCARED: Screen for Child Anxiety Related Emotional Disorders
SCAS: Spence Children’s Anxiety Scale
SCQ: Social Communication Questionnaire
SIB: Self-injurious behavior
sMRI: Structural MRI
SPPA: Self-Perception Profile for Adolescents
SRS: Social Responsiveness Scale
SRS-2: SRS 2nd Ed
SSH: Scott S. Hall, Ph.D.
SSRI: Selective serotonin reuptake inhibitor
Trofinetide: Neu-2566-FXS-001 study
VAS: Visual analog scale
VAS-C: VAS-﻿clinician rated
VAS-mb: VAS-multiple behaviors
VAS-P: VAS-parent rated
VCFS: Velo-cardial-facial syndrome
Vineland-II: Vineland Adaptive Behavior Scales 2nd Ed
vMRI: Volumetric MRI
W-ADL: Waismen Activities of Daily Living Scale
WEK: Walter E. Kaufmann, M.D.
WIAT-II: Wechsler Individual Achievement Test-II
WISC-IV: Wechsler Intelligence Scale for Children 4th Ed
WISC-R: Wechsler Intelligence Scale for Children-Revised
WISC-V: Wechsler Intelligence Scale for Children 5th Ed
WJ: Woodcock Johnston
WM: Working memory tests
WPPSI-IV: Wechsler Preschool and Primary Scale of Intelligence 4th Ed
